# Indium‐Free Recombination Layer for Perovskite‐Based Multijunction‐Solar‐Cells‐ with Improved Performance Using Sputtered Zinc Tin Oxide

**DOI:** 10.1002/smll.202511646

**Published:** 2025-11-18

**Authors:** Maryamsadat Heydarian, Georgios Loukeris, Martin Bivour, Christoph Messmer, Minasadat Heydarian, Oliver Fischer, Clemens Baretzky, Alexander J. Bett, Estelle Gevais, Muhammad Fareed U Din Masood, Sofiia Kosar, Stefaan De Wolf, Florian Schindler, Martin C. Schubert, Markus Kohlstädt, Juliane Borchert, Uli Würfel, Patricia S. C. Schulze, Andreas W. Bett, Stefan W. Glunz

**Affiliations:** ^1^ Fraunhofer Institute for Solar Energy Systems Heidenhofstr. 2 79110 Freiburg Germany; ^2^ Freiburg Materials Research Center FMF University of Freiburg Stefan‐Meier‐Str. 21 79104 Freiburg Germany; ^3^ Institute of Physics University of Freiburg Herman‐Herder‐Straße 3 79104 Freiburg Germany; ^4^ Chair for Photovoltaic Energy Conversion Department of Sustainable Systems Engineering (INATECH) University of Freiburg Emmy‐Noether‐Str. 2 79110 Freiburg Germany; ^5^ Department of Physics Technical University of Munich James‐Franck‐Str. 1 85748 Garching Germany; ^6^ Center for Renewable Energy and Storage Technologies (CREST) Physical Science and Engineering Division (PSE) King Abdullah University of Science and Technology (KAUST) Thuwal 23955‐6900 Kingdom of Saudi Arabia

**Keywords:** all‐perovskite tandem solar cell, photovoltaics, recombination layer, tandem solar cells, triple‐junction solar cell

## Abstract

Monolithic two‐terminal perovskite‐based multijunction solar cells are considered as the next generation technology in the photovoltaic market, thanks to their high power conversion efficiency at potentially low production costs. In these structures, an interlayer is required for subcell interconnection. Commonly used recombination layers are based on scarce and expensive materials such as gold or indium. Developing alternative candidates without compromising device performance is therefore crucial for the future of such multijunction technologies. Herein, low‐temperature sputtered zinc tin oxide (ZTO) is employed as an indium‐free recombination layer between two perovskite subcells. ZTO is deposited using scalable in‐line sputtering. Slight zinc doping of tin oxide is needed to enable the DC sputtering from a rotary target. Employing ZTO results in improved device performance compared to the reference devices with indium tin oxide recombination layer, deposited via a lab‐type sputtering tool. Perovskite/perovskite/silicon triple‐junction and all‐perovskite tandem solar cells show increased open‐circuit voltages by ≈60 and ≈30 mV, respectively. The work showcases that ZTO can be sputtered in a non‐destructive and scalable manner in different perovskite‐based multi‐junction technologies.

## Graphical abstract

In two‐terminal multijunction solar cells, the subcells are connected in series through recombination layers whose electro‐optical properties directly influence all photovoltaic parameters. Here, we introduce low‐temperature sputtered zinc tin oxide (ZTO) as an indium‐free recombination layer for perovskite‐based multijunction devices. Incorporating ZTO improves device performance in both perovskite/perovskite/silicon triple‐junction and all‐perovskite tandem solar cells, confirming efficient, indium‐free interconnection.

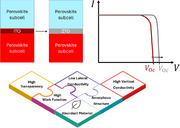

## 1 Introduction

Recently, several advancements for two‐terminal perovskite‐based tandem solar cells both in terms of power conversion efficiency (*PCE*) and upscaling have been achieved. The *PCE* of small laboratory scale perovskite/silicon and all‐perovskite tandem solar cells have surpassed 34% and 30%, respectively.^[^ ^1^ ^]^ On a larger scale, a *PCE* of 28.6% for a perovskite/silicon solar cell on an industry‐relevant M10‐sized wafer (wafer size of 182 mm × 182 mm) has been reported by Qcells^[^ ^2^ ^]^ and a *PCE* of 26.9% is reported for a 60‐cell residential‐size module, produced by Oxford PV.^[^ ^3^ ^]^ Motivated by such recent advancements, new research targets even higher performances by combining both technologies to develop perovskite/perovskite/silicon triple‐junction solar cells.^[^ ^4^ ^]^

Among the different multijunction solar cell configurations, a two‐terminal design allows integration with existing module and wiring design. In monolithic perovskite‐based two‐terminal multijunction solar cells, the subcells are connected in series through a recombination layer (RL). In dual‐junction solar cells, one RL is needed to connect the two subcells, while in a triple‐junction structure an additional RL is needed to interconnect the third subcell. In this regard, for efficient two‐terminal multijunction cells, optimization of the RL is crucial to minimize electrical and optical losses. This is not a trivial task, as this layer must meet several key requirements, including:

1) Material and interface properties that align with the surrounding charge transport layers (CTLs) to ensure excellent transport of majority charge carriers to RL and avoid the accumulation of charges at the interface and recombination within the subcells.
2) Minimal optical parasitic absorption, which requires a low extinction coefficient (k), and minimal reflection losses at the interfaces, which can be achieved by matching the refractive index (n) and thickness with the surrounding layers.
3) Deposition process without invoking damage to the underlying perovskite subcell, e.g. requiring a sufficient low temperature and no sputter damage.
4) Smooth and uniform surface characteristics to ensure high‐quality morphology and proper layer formation of the overlying materials. This is especially critical considering that the formation of CTL of the overlaying subcell shows a pronounced dependence on underlying surface properties.^[^ ^5^ ^]^
5) Composition from sustainable materials, with deposition methods that are compatible with large‐scale manufacturing to ensure commercial feasibility.

Finally, when solvents are involved in the processing of the perovskite on the RL, it needs to be protective against solvent penetration to avoid damaging the underlying perovskite. However, in most cases the RL is structured and therefore other layers in the structure (CTL or buffer layer) serve as the solvent barrier layer.

So far, the most common materials that are used as the RL between two perovskite subcells are either ultrathin metal layers such as gold^[^ ^6^, ^7^ ^]^ or indium‐based transparent conductive oxides (TCOs).^[^ ^7^, ^8^ ^]^ However, employing gold as RL comes with optical disadvantages. It has been shown, that even an ultrathin layer of gold introduces notable optical parasitic absorption that limits the amount of light transmitted to the other subcells.^[^ ^8^, ^9^ ^]^ Although indium‐based TCO RLs offer superior optical properties,^[^ ^8^, ^9^, ^10^, ^11^ ^]^ due to the scarcity and high costs of indium, their use in solar cell mass production poses scalability challenges for the perovskite‐based solar cell technology at a Terawatt (TW) level.^[^ ^12^ ^]^ One approach to address these concerns is to omit the RL and to directly contact the CTLs of the two perovskite subcells. Zhou et al. demonstrated such a solar cell by directly depositing the hole transport layer (HTL) of the low‐bandgap (LBG) subcell (poly(3,4‐ethylenedioxythiophene) polystyrene sulfonate, PEDOT:PSS) onto the electron transport layer (ETL) (tin oxide, SnO*_x_*) of the high‐bandgap (HBG) subcell.^[^ ^13^ ^]^ Yu et al. fabricated an all‐perovskite tandem solar cell without a RL by replacing the PEDOT:PSS with SnO*_x_* as HTL of the LBG perovskite. To achieve this, they modified the composition of SnO*_x_*, deposited via atomic layer deposition (ALD) to SnO_1.76_ that has shown to have ambipolar carrier transport properties.^[^ ^14^ ^]^ However, transferability of this strategy to replace other HTLs is not tested so far. In addition, several studies have shown that the large work function difference between the ETL of the bottom cell and the HTL of the top cell acts as a barrier to carrier transport, which results in an S‐shape in the current density–voltage (*jV*) curve for RL‐free devices.^[^ ^10^, ^11^, ^15^ ^]^ To mitigate this issue a very thin layer of TCO has been shown to be necessary to reduce the work function mismatch, facilitating the transport and provide recombination sites for the charge carriers to achieve a high fill factor (*FF*).^[^ ^10^, ^11^, ^15^ ^]^ However, no demonstration of successful implementation of an indium free TCO between two perovskite subcells has been reported to date.

Here, we report a low‐temperature sputtered zinc tin oxide (ZTO) as an alternative indium‐free RL between two perovskite subcells. Earlier, sputtered ZTO layers have been introduced in silicon solar cells as an alternative to Aluminum doped zinc oxide.^[^ ^16^ ^]^ Furthermore, it has been developed and used as anode in organic light‐emitting diodes by Morales‐Masis et al.^[^ ^17^ ^]^ In another study, an ALD deposited bilayer of SnO*_x_*/ZTO has been employed as a buffer layer to prevent the sputter damage during the sputtering of the top TCO layer.^[^ ^18^, ^19^ ^]^ A similar approach was used in the fabrication of all‐perovskite tandem solar cells where indium tin oxide (ITO)Ultraviolet serves as the RL, and an ALD deposited SnO*_x_*/ZTO bilayer was used as a buffer layer.^[^ ^20^ ^]^ ZTO has also been employed as an ETL in organic solar cells^[^ ^21^ ^]^ where the ZTO layer was formed by spin‐coating. A high‐temperature annealing step of 500 °C was required, which was shown to be necessary in the case of solution processed ZTO. So far, there has only been one report on using sputtered ZTO as a RL between silicon and perovskite.^[^ ^22^ ^]^ In that work, an *n‐i‐p* perovskite solar cell was used and a high annealing temperature step of 500 °C was found helpful to improve the electrical and optical properties of the ZTO layer. In addition, the thickness of the ZTO had a strong effect on the optical interference pattern in the external quantum efficiency (EQE) of the bottom cell. Therefore, an optimum layer thickness of around 150 nm was needed to increase the light coupling in the silicon bottom cell. However, to employ this layer as a RL between two perovskite subcells, first, an annealing step of more than 100 °C likely induces heat damage to the underlying perovskite absorber. Second, it is known that thicker layers increase substantially optical parasitic absorption and therefore are not suitable toward efficient tandems.

In this work, for the series connection of two perovskite subcells, we employ a thin single layer of ZTO. The ZTO is DC sputtered with no active heating using a rotary target with a nominal composition of (SnO_2_/ZnO = 92 /8 wt.%). A main motivation for adding a slight ZnO fraction to the SnO*_2_* host material was to obtain sufficient electrical conductivity of the target to enable good sputtering conditions using a DC power supply and not rely on RF sputtering. The choice of an in‐line tool over a lab‐type static tool and the use of a rotary cylindrical target over a planar target was also motivated by meeting the standards of industrial sputter deposition of metal oxides. We compare the electrical, optical, and structural properties of the as‐deposited 15 nm ZTO with no post annealing to a 15 nm ITO layer. The developed layer is implemented as RL between two perovskite subcells in both all‐perovskite tandem and perovskite/perovskite/silicon triple‐junction structures. Different HTLs (PEDOT:PSS and [2‐(9*H*‐carbazol‐9‐yl)ethyl]phosphonic acid (2PACz)) and perovskites (HBG lead‐based and LBG lead‐tin‐based) have been processed on the ZTO layer to investigate the compatibility of the layer with a wide range of materials and processing conditions. Finally, by employing advanced characterization techniques and simulation, we identify the possible origins of the open‐circuit voltage (*V*_OC_) improvement in ZTO‐based devices.

## 2 Results and Discussion

### 2.1 Analysis of the Zinc Tin Oxide Layer

**Figure 1:**
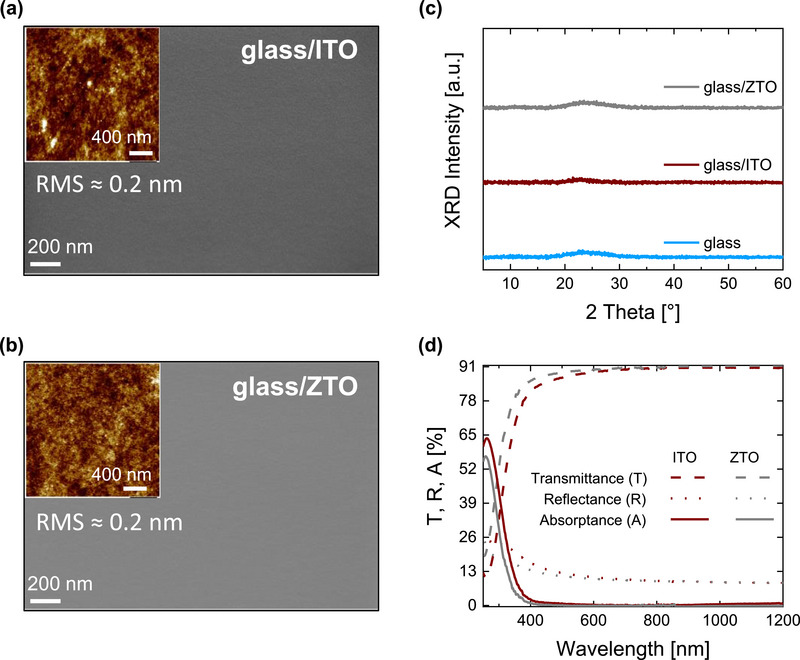
a) Top view SEM images with an AFM topography inset of 15 nm ITO and b) 15 nm ZTO. c) XRD patterns of glass substrate, ITO, and ZTO layers. d) Transmittance, reflectance, and absorptance spectra of ITO and ZTO layers.

The ZTO layer is deposited using an industry‐compatible sputter process (in‐line DC sputtering, ≈600 mm long rotary target, power density ≈4.8 W cm^−^^2^ [1.5 kW], and a mixture of O_2_ and Ar as process gas with grade 6.0 purity) with no post treatment. The properties of the ZTO film are compared to a reference structure consisting of 15 nm sputtered ITO. The ITO was deposited using a lab‐type process (static DC sputtering, ≈254 mm ∅ planar target (In_2_O_3_/SnO_2_ = 90/10 wt.%), power density ≈0.08 W cm^−^^2^ [40 W]). From atomic force microscopy (AFM) and scanning electron microscopy (SEM) measurements, both layers show a homogeneous closed film with 0.20 nm root mean square (RMS) (**Figure** **1**a,b), in line with what has been previously reported forsputtered ZTO.^[^ ^17^ ^]^ In addition, the X‐ray diffraction (XRD) analysis shows no diffraction peaks, confirming that both materials have an amorphous structure (Figure 1c). We note that the as‐deposited ITO film shows an amorphous structure. Our ITO only shows a polycrystalline structure after annealing at 300 °C for 5 min, while ZTO remains amorphous (Figure S1, Supporting Information).

In Figure 1d the optical properties of the 15 nm ZTO layer are compared to the 15 nm of ITO film. ZTO shows excellent transparency, more than 90% of the incident radiation from 620 to 1200 nm. Both films show negligible absorption in the wavelength range between 500 and 1200 nm that corresponds to the spectral range where the lower bandgap subcells absorb. The ZTO shows a slightly higher bandgap (0.1 eV) compared to ITO (see Figure S2, Supporting Information). To identify the charge carrier mobility, doping density and sheet resistance, hall measurements were performed on ITO and ZTO layers (**Table** **1**). The measurements were conducted on 50 nm thick layers. Kelvin probe (KP) measurements (Table 1) revealed a higher work function for ZTO compared to ITO. This could be associated to the lower carrier density of ZTO, which results in a shift of the Fermi level toward mid‐gap and hence closer to the valence band.^[^ ^23^ ^]^

**Table 1:** Summary of the Hall parameters on 50 nm ZTO and ITO layers together with the work function of ITO and ZTO layers determined from kelvin probe (KP) measurements on 15 nm ITO and ZTO.

Material	Mobility	Doping	Sheet resistance	Material type	Work function
	[cm^2^ (V s)^−1^]	[1 cm^−^^3^]	[Ω/□]		[eV]
ZTO	15.5	0.10 × 10^20^	7740	n‐type	5.40
ITO	30.2	5.09 × 10^20^	81.4	n‐type	4.85

Additionally, to assess if the higher resistivity of the ZTO layer poses a fundamental limitation to its vertical conductivity, architectures glass/ITO/ITO/Ag and glass/ITO/ZTO/Ag were fabricated. A reference structure with a glass/ITO/Ag architecture was also included to identify the baseline contact resistance. As shown in Figure S3 (Supporting Information), the ZTO and ITO layers exhibit only a marginal difference in resistance from 37.3 to 40.7 mΩ, comparable to the test structure without any intermediate layer.

### 2.2 Implementation in Monolithic Two‐Terminal MultiJunction Solar Cells

**Figure 2:**
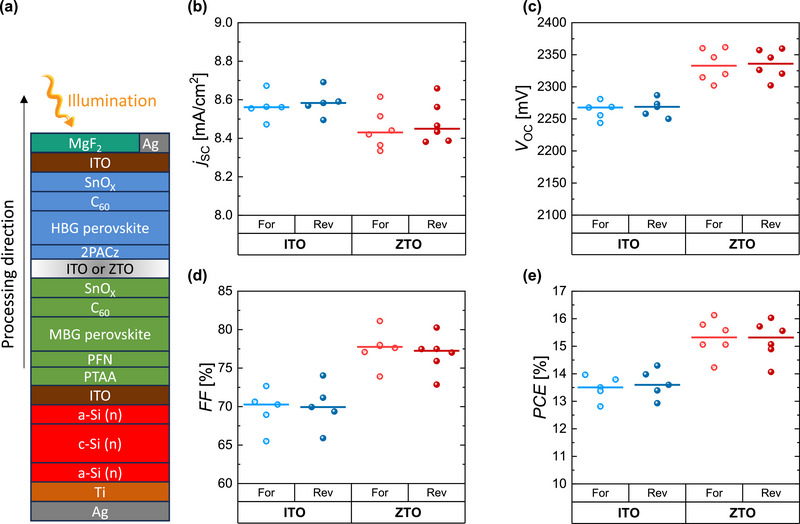
a) Schematic of the MBG perovskite/HBG perovskite dual‐junction solar cell structure on an ohmic silicon substrate. The illumination and processing directions are shown with yellow and black arrows, respectively. b–e) the photovoltaic parameters of the devices with ITO and ZTO as recombination layer between perovskite subcells.

We compared the performance of multijunction solar cells with ITO or ZTO as recombination layer between the perovskite subcells. First, we implemented the ZTO layer as a RL between the perovskite top and middle cells in perovskite/perovskite/silicon triple‐junction solar cells. Here, the ZTO is deposited on the middle‐bandgap (MBG) perovskite (1.56 eV); and the HBG (1.83 eV) subcell is processed on top. Spin‐coated 2PACz is used as an HTL of the top cell, whose formation has been shown to be dependent on its underlying surface.^[^ ^5^, ^24^ ^]^ To test the compatibility of the RL with 2PACz, we first fabricated MBG perovskite/HBG perovskite dual‐junction solar cells to evaluate the RL without the influence of the silicon subcell. For this purpose, we used ohmic silicon substrates (i.e., silicon substrates lacking the p/n‐junction, more information can be found in the material method section) to mimic the structure, design, and processing of the triple‐junction solar cells. Solar cells with the structure silicon substrate/ITO/poly‐[bis‐(4‐phenyl)‐(2,4,6‐trimethylphenyl)‐amin](PTAA)/poly[(9,9‐bis(3′‐(N,N‐dimethylamino)propyl)‐2,7‐fluorene)‐alt‐2,7‐(9,9‐dioctylfluorene)‐bromide](PFN‐Br)/MBG perovskite/C_60_/SnO*_x_*/RL/2PACz/HBG perovskite/C_60_/SnO*_x_*/ITO/Ag/MgF_2_ (**Figure** **2**a) with an active area of 1 cm^2^ were fabricated. The photovoltaic parameters of the dual‐junction solar cells using either ITO or ZTO as RL are presented in Figure 2b–e and the *jV* curves of the champion ITO and ZTO containing devices are presented in Figure S4 (Supporting Information). These devices are measured with adjusted spectrum according to.^[^ ^25^ ^]^ Details on measurements can be found in the material and method section.

**Figure 3:**
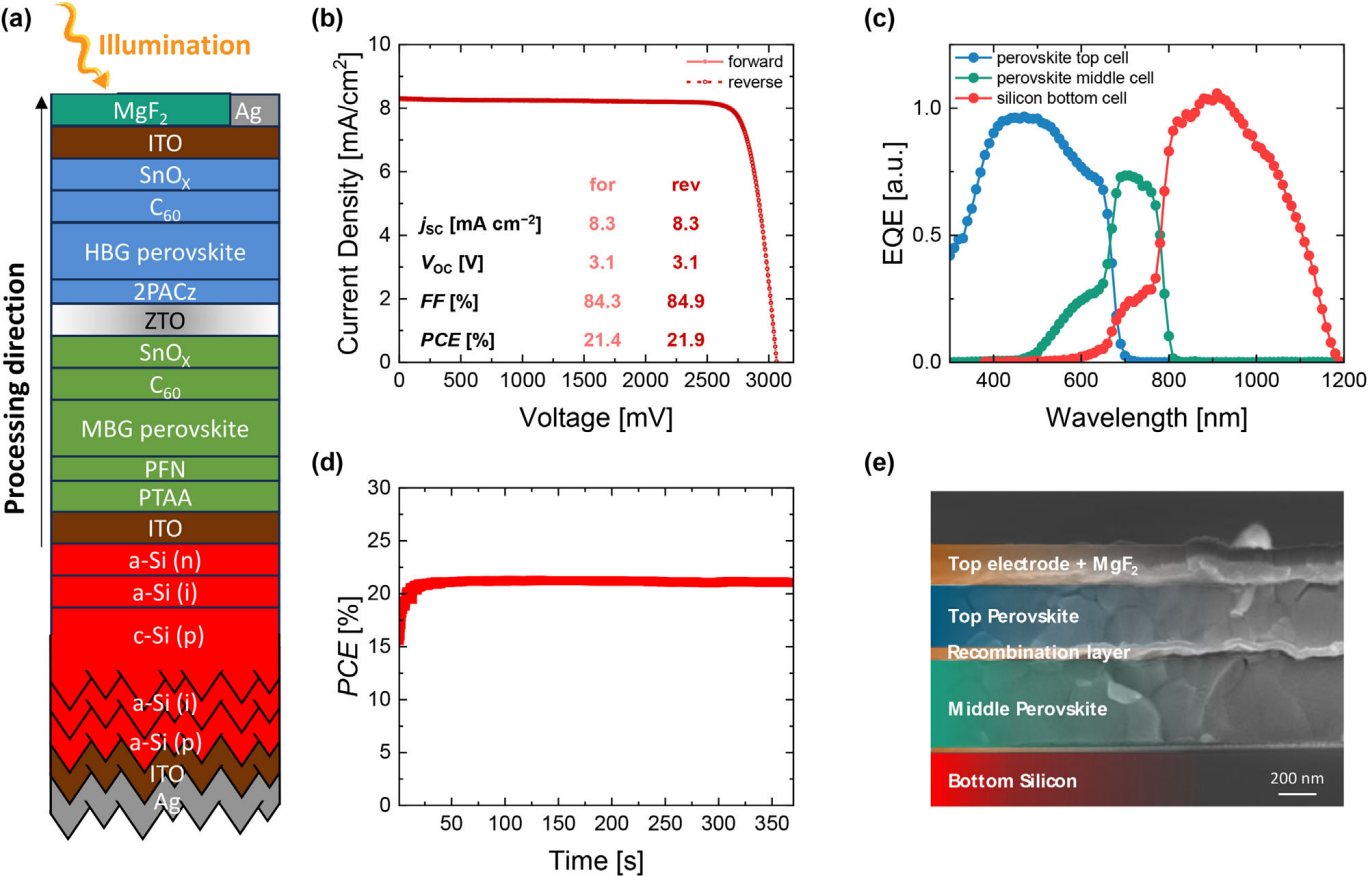
a) Schematic of the perovskite/perovskite/silicon triple‐junction device structure. The illumination and processing directions are shown with yellow and black arrows, respectively. b) *jV* curve, c) EQE curves, d) efficiency of the champion triple‐junction solar cell measured over time at fixed voltage close to maximum power point and e) the cross‐section SEM image of a triple‐junction solar cell with ZTO recombination layer.

The group with ZTO as RL shows improved performance compared to the reference ITO‐based group due to better *FF* and *V*_OC_. The average *V*_OC_ improvement is ≈70 mV, while *FF* improvements reach up to 9%. Further discussion on the origin of the improvements will take place in the following. To evaluate if the ZTO impacts the photostability of the HBG perovskite, PL measurement of HBG single‐junction solar cell on ITO or ZTO was performed over an extended period of time. From Figure S5 (Supporting Information), ZTO does not offer any significant advantages nor disadvantages compared to ITO in this regard. The HBG perovskite on ZTO shows marginal red shift of the PL peak, whereas on ITO a second PL peak at lower wavelength appears. Hence, both samples exhibit same degree of stability. We then fabricated perovskite/perovskite/silicon triple‐junction solar cells on a silicon heterojunction bottom cell with flat front side and textured rear side (**Figure** **3**a). EQEs of the dual‐junction and triple‐junction solar cells with ITO and ZTO RL are shown in Figure S6 (Supporting Information). Even though the EQEs are not absolutely calibrated, their similar shape confirms that optically the ZTO and ITO have similar absorption behavior. Statistical photovoltaic parameters of the triple‐junction solar cells with ITO and ZTO as RL between the two perovskite subcells are presented in Figure S7 (Supporting Information). Similarly, triple‐junction solar cells with ZTO as RL show ≈60 mV improvement in *V*_OC_. We note that only 5 nm ITO was used for the reference samples here. However, triple‐junction devices with 5 and 15 nm ITO showed comparable performance (Figure S8, Supporting Information). The champion triple‐junction solar cell with a ZTO RL reached 21.9% *PCE*, with a *j*_SC_ of 8.3 mA cm^−2^, *FF* of 84.9% and a *V*_OC_ of 3.1 V in reverse scan direction (Figure 3b). This is among the highest *V*_OC_ values reported in literature for the perovskite/perovskite/silicon triple‐junction structure (Figure S9, Supporting Information). The *PCE* evolution over 5 min at a fixed voltage close to the maximum power point voltage is shown in Figure 3d. Due to the still nonoptimized bandgaps and thicknesses of the perovskite absorbers (Figure 3c,e), the overall current density is limited by the middle cell.

To evaluate the stability of the devices with the ZTO RL, an unencapsulated device was stressed at a voltage close to MPP in ambient conditions (25 °C, ≈60% relative humidity) under 1‐sun illumination for a longer time. Figure S10a (Supporting Information) shows that around 90% of the power output was maintained for 10 h of measurement time. However, due to the relatively high humidity, after the measurement, the sample showed signs of degradation (yellow spot appeared on the active area as shown in Figure S10b, Supporting Information). To gain insight into the operational stability excluding the humidity factor, we enclosed the device into a chamber, where humidity was reduced to 20% by flushing with N_2_ (see Figure S10c, Supporting Information). In the absence of the humidity, our unencapsulated sample retained 98% of its maximum efficiency for 60 h as shown in Figure S10d (Supporting Information).

To verify the universality of the ZTO as an effective RL between perovskite subcells, all‐perovskite tandem solar cells consisting of a LBG absorber with a bandgap of 1.22 eV and a HBG with bandgap of 1.77 eV were built. The HTL of the LBG subcell deposited onto the ZTO is processed by spin‐coating PEDOT:PSS. The solar cells are made in a superstrate configuration, deposited on glass, having a cell area of 0.0925 cm^2^. It is important to mention that the order of processing is different in all‐perovskite tandem opaque cell processed on a glass to a perovskite/perovskite/silicon triple‐junction cell. In all‐perovskite tandem the ZTO is deposited onto the HBG perovskite and the LBG perovskite is processed on top, contrary to what has been done in the previous section where the HBG perovskite is deposited onto the ZTO layer.

**Figure 4:**
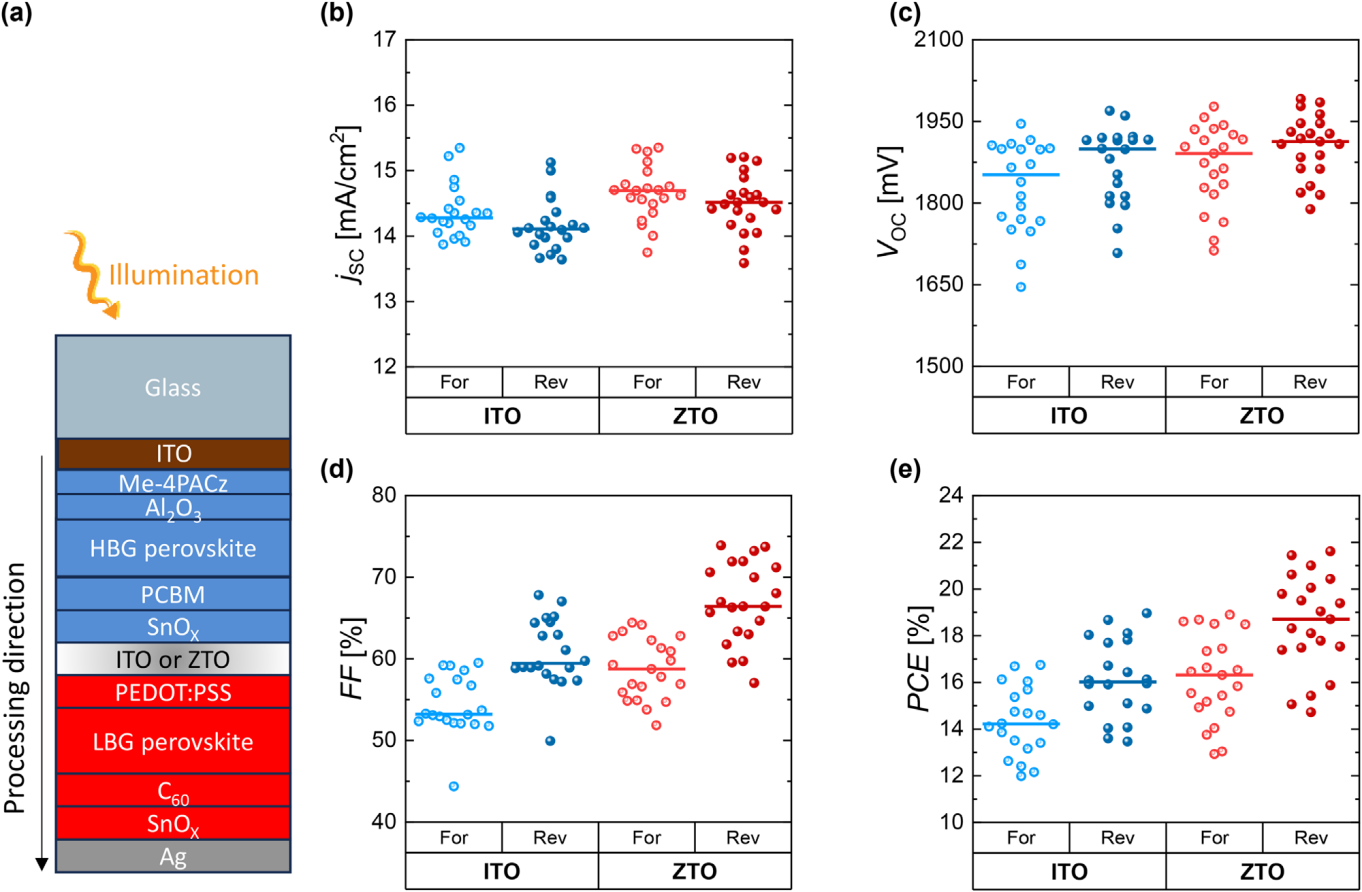
a) Schematic of the all‐perovskite tandem solar cell structure. The illumination and processing directions are shown with yellow and black arrows, respectively. b–e) The photovoltaic parameters comparing ITO to ZTO as recombination layer between the perovskite subcells. The measurements have been carried out without spectral adjustment.

The solar cells consist of glass/ITO/ [4‐(3,6‐dimethyl‐9*H*‐carbazol‐9‐yl)butyl]phosphonic acid (Me‐4PACz)/aluminum oxide (Al_2_O_3_)/HBG perovskite/[6,6]phenyl‐C_61_‐butyric acid methyl ester (PCBM)/SnO*_x_*/RL/PEDOT:PSS/LBG perovskite/C_60_/SnO*_x_*/Ag as can be seen in **Figure** **4**a. Photovoltaic parameters of all‐perovskite tandem solar cells featuring ITO or ZTO as RL are shown in Figure 4b–e. Consistent with the trend observed in the triple‐junction solar cells, devices with ZTO as RL show enhanced *V*_OC_ compared to ITO‐based devices with an average *V*_OC_ increase of 30 mV in both forward and reverse scan direction. Further insights to identify the origins of the observed *V*_OC_ increase for all multijunction layouts will be given in the following section. It is important to note that these devices were measured with a single source solar simulator. Thus, the spectrum was not adjusted based on the spectral responses of the subcells and therefore *j*_SC_ and *FF* values have large uncertainties. The relative EQEs show slight differences in the bottom cell (Figure S11, Supporting Information). The *jV* curves of the champion solar cells from both groups are presented in Figure S12 (Supporting Information) that also show a *V*_OC_ increase of ≈30 mV. The device with ZTO recombination layer was also stressed at a voltage close to MPP in ambient conditions (25 °C, ≈60% relative humidity) under 1‐sun illumination for a longer time. Note that the sample was not encapsulated. From Figure S13 (Supporting Information) close to 90% of the power output was maintained for 20 h of measurement time.

### 2.3 Origin of the *V*_OC_ Improvement in Devices with ZTO Recombination Layer

To understand the origin of the *V*_OC_ improvement in the multijunction solar cells featuring ZTO as RL, we conducted subcell selective photoluminescence (PL)‐based implied open‐circuit voltage (i*V*_OC_) imaging.^[^ ^26^ ^]^ This technique was utilized for both, all‐perovskite tandem solar cells as well as on perovskite/perovskite/silicon triple‐junction solar cells. Later, the respective averaged global i*V*_OC_ values of the individual subcells are compared with the *V*_OC_.

**Figure 5:**
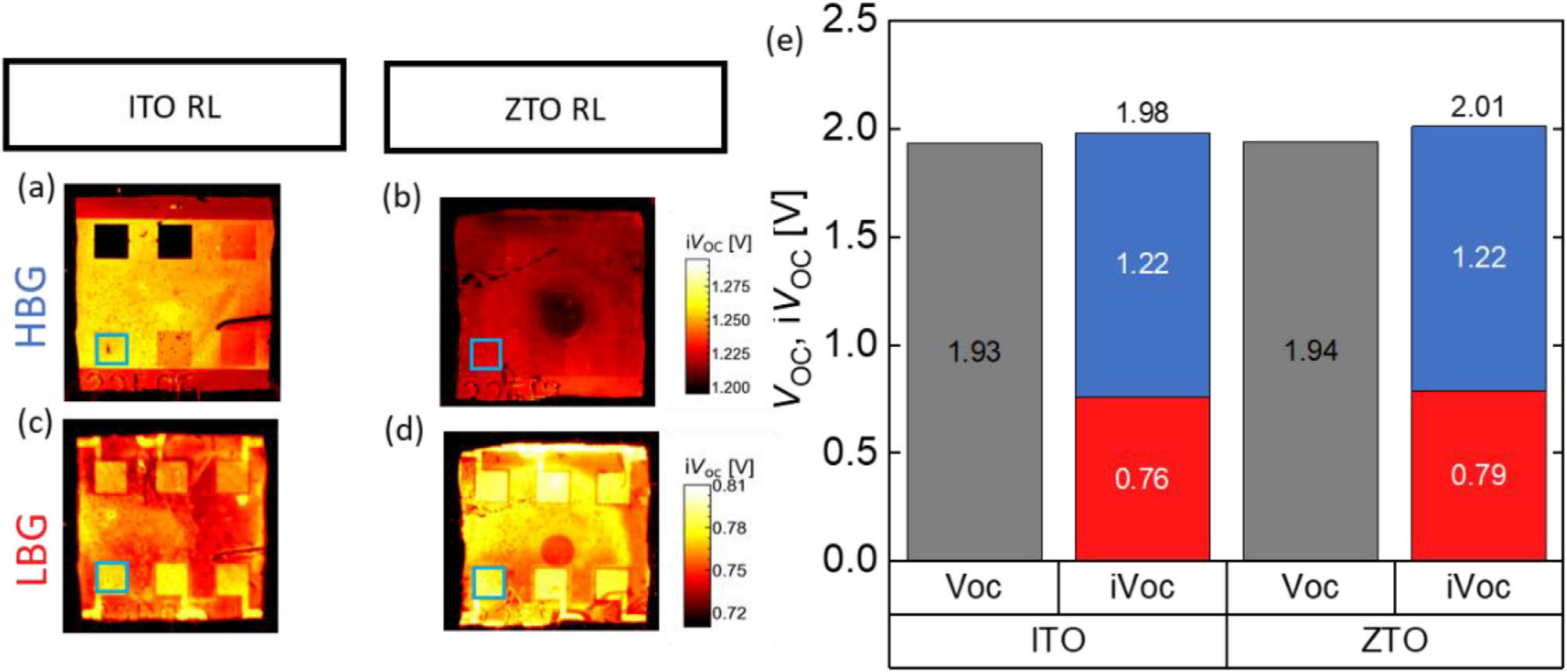
Subcell selective *iV*_OC_ images of all‐perovskite solar cells with ITO or ZTO recombination layer. a,b) correspond to the HBG subcells and c,d) to the LBG subcells. Enclosed with the blue square the investigated solar cell is depicted. e) Subcell *iV*_OC_ values (red and blue for LBG and HBG, respectively) of chosen solar cells for ITO and ZTO are shown and compared to the corresponding device *V*_OC._ The selectivity loss (*iV*_OC_−*V*_OC_) is 30 and 7 mV for sample with ITO and ZTO RLs, respectively.

First, for the case of the all‐perovskite tandem solar cells, the i*V*_OC_ images of individual subcells for each group are shown in **Figure** **5**a–d. For the champion solar cells of each group, the i*V*_OC_ mean values of LBG and HBG subcells, along with the measured *V*_OC_ can be seen in Figure 5e. The RL marginally affects the i*V*_OC_ of the HBG absorber, because the absorber serves as the bottom cell (in processing sequence) and is already coated before the RL deposition. The similar i*V*_OC_ of the HBG for ZTO containing samples indicates no sputter damage to the ETL and subcell underneath, even though a much higher power density is used compared to the lab‐type ITO deposition (4.8 W cm^−^^2^ vs 0.08 W cm^−^^2^). Additionally, underlying the RL a 30 nm ALD‐deposited SnO*_x_* layer and a 20 nm spin‐coated PCBM layer serve as ETLs, and the LBG perovskite, is deposited onto a 20 nm PEDOT: PSS layer, which is spin‐coated onto the RL. In contrast to thick ETLs, for such thin HTL layers, the electronic properties of the RL influence the selectivity of the RL that leads into boosting the tandem device *V*_OC_.^[^ ^27^ ^]^ Overall, from Figure 5c,d it is shown that utilizing ZTO leads to a decrease in the non‐radiative recombination in the LBG perovskite subcell, leading to higher i*V*_OC_. The 30 mV improvement in the i*V*_OC_ of the LBG absorber directly transfers to the *V*_OC_ improvement of the all‐perovskite solar cells shown in Figure 4c.

**Figure 6:**
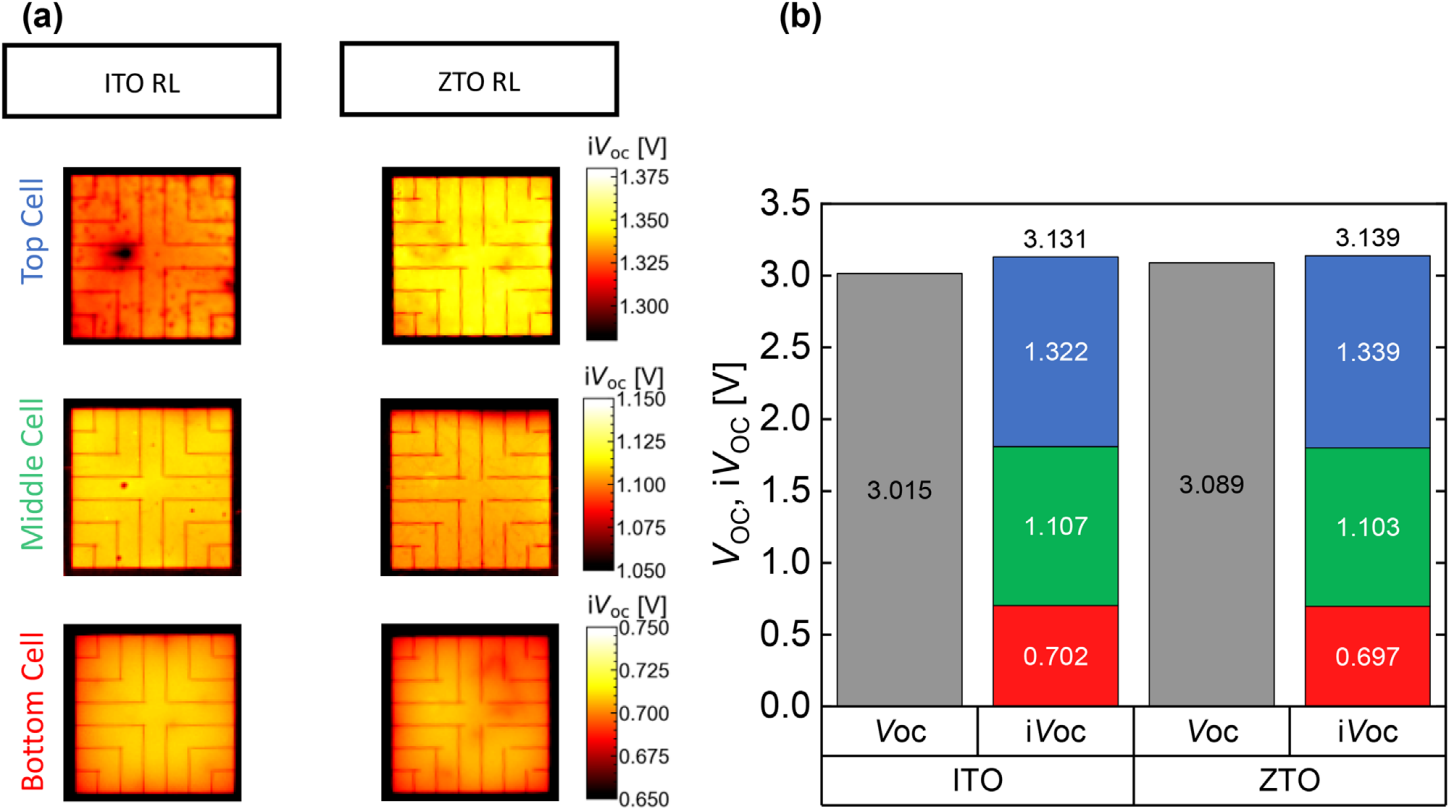
a) Subcell selective *iV*_OC_ images of triple‐junction solar cells with ITO or ZTO recombination layer and b) respective averaged global *iV*_OC_ values of the individual subcells (red, green, and blue for silicon cell, MBG and HBG cells, respectively) together with the V_OC_ of the devices. The selectivity loss (*iV*_OC_−*V*_OC_) is 116 and 50 mV for the samples with ITO and ZTO RLs, respectively.

Similarly, for the case of the perovskite/perovskite/silicon triple‐junction solar cells, i*V*_OC_ images of the individual subcells for devices with ITO or ZTO RL are shown in **Figure** **6**a. The comparison of i*V*_OC_ to *V*_OC_ of the devices is shown in Figure 6b. The i*V*_OC_ of the silicon bottom and middle perovskite cells are close, within the measurement uncertainty. The top cell processed on ZTO however, shows a 17 mV higher i*V*_OC_ than the top cell on ITO as RL that is not very significant. Moreover, it is also an indication that there are no additional recombination active defects at ZTO/2PACz interface compared to ITO/2PACz interface. Here also, the similar i*V*_OC_ of the middle cell with ZTO and ITO as RL is an indication that there was no damage induced by sputter process in any underlying layer. This confirms the compatibility of the scalable high‐power deposition method with our solar cell fabrication process. Overall, the sum of the i*V*_OC_ of the three subcells in the sample with ZTO RL is not significantly higher than the sample with ITO that is not in line with the >50 mV higher *V*_OC_ of this group. The same trend is observed when measuring different devices (shown in Figure S14, Supporting Information).

This is different from the case of all‐perovskite solar cells where the LBG perovskite with PEDOT:PSS HTL was processed on the RL. So, there should be other sources of improvement for the *V*_OC_ upon replacing the ITO with ZTO in this case.

### 2.4 Simulation

To understand the origin of the *V*_OC_ improvement in all‐perovskite tandem and perovskite/perovskite/silicon triple‐junction solar cells, a simulation study was performed using Sentaurus TCAD, based on the model described in Messmer et al.^[^ ^28^ ^]^ Our initial focus is on the perovskite/perovskite/silicon triple‐junction solar cell, utilizing the simulation framework presented in Restat et al.^[^ ^29^ ^]^ 2PACz is used as HTL for this architecture. Hence, the goal is to elucidate fundamental impact factors for the perovskite/self‐assembling monolayer (SAM)/TCO heterojunction contact system.

**Figure 7:**
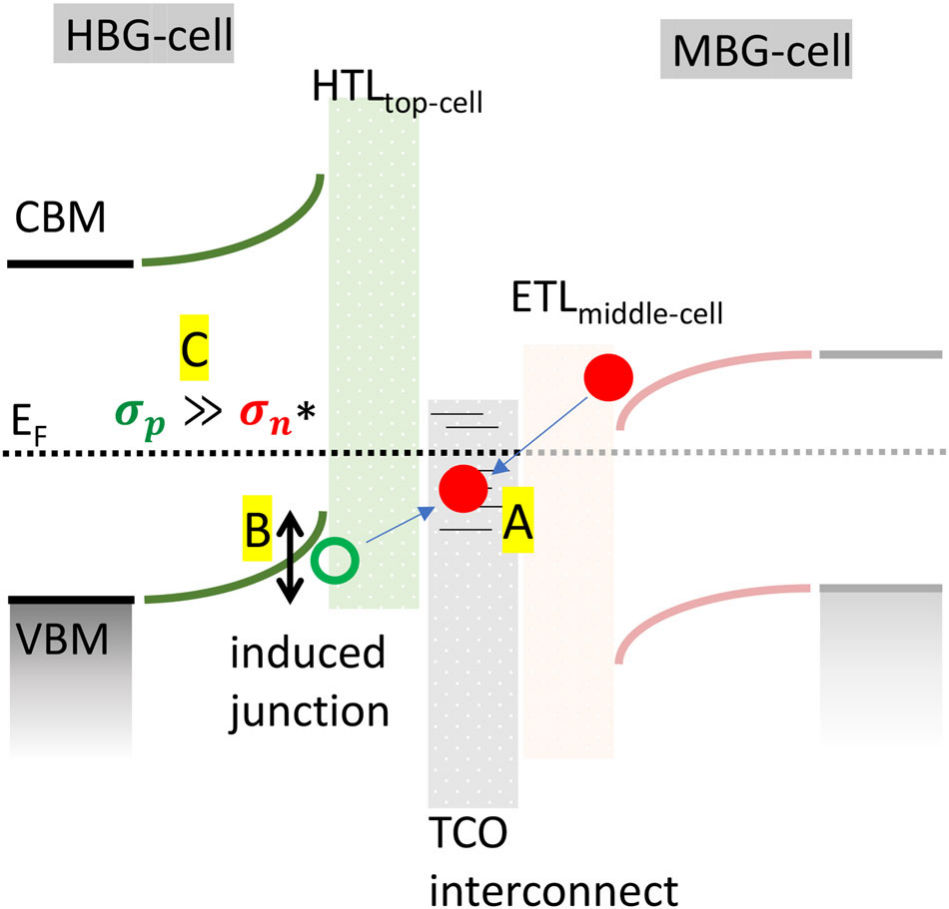
Simplified band diagram of subcell interconnection region using a TCO interlayer between HTL of the HBG perovskite and ETL of the MBG perovskite. A) current flow from ETL to HTL via desired trap‐assisted recombination. B) band bending induced in the HBG perovskite absorber. C) Undesired minority charge carrier recombination due to poor transport of holes to the RL.

**Figure** **7** presents the simplified band diagram of the interconnection of HBG and MBG perovskites featuring 2PACz as HTL of the HBG subcell. The fundamental mechanism of transport of charge carriers from the respective subcells to the TCO and their recombination is shown. A) Indicates the current flow from ETL to HTL via desired trap assisted majority carrier recombination of electrons and holes in bulk / interface defect states of the TCO. B) Indicates the band bending induced in the HBG perovskite absorber as a result of the combined HTL and TCO interconnection properties due to the effective work function of the TCO after SAM deposition. Basically, a high band bending is desirable to ensure a large hole conductivity of this induced junction to facilitate efficient hole extraction, low recombination, and low selectivity losses. C) The holes that are not transported to the TCO accumulate at the interface, which in turn increases the probability of undesired minority charge carrier recombination in the hole contact region.

**Figure 8:**
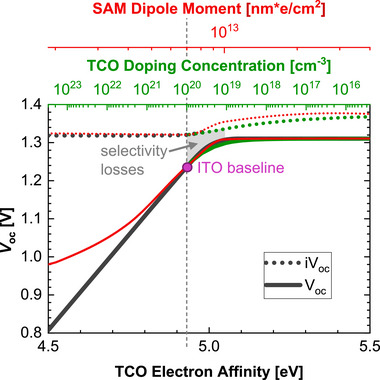
Open‐circuit voltage (*V*_OC_) of the HBG perovskite subcell as a function of TCO electron affinity, TCO doping concentration, and SAM dipole moment, whereby the ITO baseline is the standard set of simulation parameters and only the parameter on the respective *x*‐axis is changed. Internal voltage (i*V*_OC_) is presented as dashed lines.

**Figure** **8** illustrates *V*_OC_ of the HBG perovskite subcell as a function of three essential TCO parameters, those being electron affinity (black axis), the TCO doping concentration (green axis), and SAM dipole moment (red axis) displayed as the *x*‐axes. The baseline for the simulation is defined for an electron affinity of 4.93 eV, a doping concentration of 10^20^ cm^−3^ and a SAM dipole moment of 7 × 10^12^ nm·e cm^−^^2^. The values are based on the simulation in the work of Landgraf et al.,^[^ ^30^ ^]^ which is highlighted as “ITO baseline” (purple dot). For simplicity optical parameters are not considered in the simulation.

According to the principles of Schottky barrier formation, the built‐in potential increases in the subcell with the work function (slope of 1), which is altered by the TCO electron affinity (black x‐axis). This relationship is evident in the linear rise of *V*_OC_ (black solid line) as the TCO electron affinity ranges from 4.5 to 5.0 eV. Beyond this range, *V*_OC_ saturates around 1.3 V, representing the maximum achievable for this perovskite absorber.

As a second variation, the TCO n‐doping concentration was varied (shown on the top x‐axis in green). The *V*_OC_ as a function of the TCO doping concentration is depicted by the green solid line, beginning at 10^20^ cm^−3^. A lower doping concentration proves to increase the *V*_OC_ (and also the fill factor, not shown), as it shifts the Fermi level of the TCO closer to the valence band, thereby increasing the effective work function of the SAM/TCO contact. The axis scaling was chosen to align with the black solid line, demonstrating that changes in either electron affinity or doping concentration similarly impact *V*_OC_ by altering the effective work function.

As a third parameter, the impact of the effective dipole moment of the SAM layer (red x‐axis) is addressed. The effect on *V*_OC_ is indicated by the red solid line. The dipole moment alters the electrostatic potential at the HTL/TCO interface, increasing the effective work function with increased dipole moment.^[^ ^27^ ^]^ The scaling of this axis was selected to illustrate the interplay of these three parameters and showcase the beneficial effects on the HBG subcell *V*_OC_. The effect of the SAM dipole moment vanishes at very low values, and the effect of the TCO work function (for this variation fixed to 4.8 eV) becomes visible.

In addition, the internal voltage (i*V*_OC_) is presented as dashed lines for all conditions, at ≈1.32 V for a low effective work function and 1.37 V for a high effective work function. This voltage difference can be attributed to changes in perovskite bulk recombination. On the left side of the graph, a balanced electron and hole concentration within the perovskite leads to increased Shockley–Read–Hall (SRH) bulk recombination. Conversely, with a high effective work function (TCO/SAM), bulk behavior is dominated by injected holes from the HTL, which effectively suppresses SRH recombination. The higher WF of ZTO/SAM compared to ITO/SAM is confirmed by the KPFM measurement. In addition, the work function increase upon SAM deposition is more pronounced on ZTO, suggesting a larger effective dipole moment (Figure S15, Supporting Information).

Selectivity losses, defined as the difference between internal and external voltage^[^ ^31^ ^]^ are highlighted in gray within the region of interest. For instance, transitioning from 10^20^ to 10^19 ^cm^−3^ in doping concentration, as observed for ITO and ZTO, respectively, accounts for the slight increase in i*V*_OC_ and significant rise in *V*_OC_, thereby reducing selectivity losses that is in line with the experimental observations.

**Figure 9:**
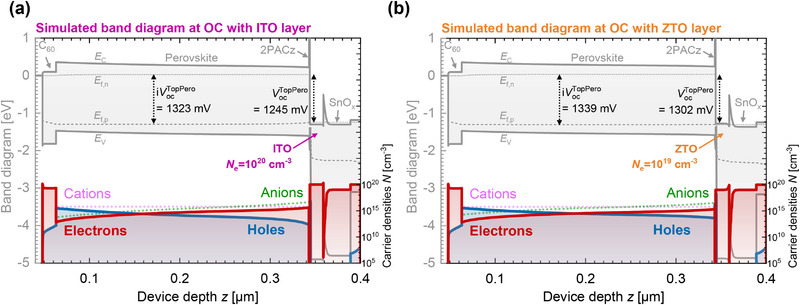
Simulated band diagrams of the perovskite top cell under open‐circuit condition. a) The band diagram with an ITO layer exhibiting a high electron concentration of 10^20^ cm^−3^, showing depletion of holes within the perovskite and selectivity losses evidenced by the difference between *V*_OC_ and iV_OC_. b) The band diagram with a ZTO layer at a doping concentration of 10^19^ cm^−3^, indicating improved hole density and conductivity toward the HTL, resulting in higher V_OC_ and slightly improved iV_OC_ with reduced selectivity losses.

**Figure** **9**a displays the simulated band diagram (left axis) of the perovskite top cell under open‐circuit (OC) condition with an ITO layer featuring a high electron concentration of 10^20^ cm^−3^. The right axis shows the densities of electrons, holes, anions, and cations within the absorber. The high ITO electron concentration (resulting in a low effective work function) causes a depletion of holes in the perovskite near the HTL contact, leading to poor hole conductivity and a visible gradient in the quasi‐Fermi level of the valence band, resulting in a smaller *V*_OC_ (1245 mV) compared to i*V*_OC_ (1323 mV), indicative of selectivity losses.

Figure 9b presents the simulated band diagram (left axis) of the perovskite top cell under OC condition with a ZTO layer exhibiting a doping concentration of 10^19^ cm^−3^, i.e., an order of magnitude lower than in the case of ITO. The lower electron concentration in ZTO (and thus higher effective work function) results in a greater hole density within the perovskite toward the HTL, as compared to the ITO case. This improvement in hole conductivity leads to a smaller gradient in the quasi‐Fermi level of holes, resulting in a significantly higher *V*_OC_ (1302 mV) and a slightly improved i*V*_OC_ (1339 mV). Consequently, selectivity losses are diminished. These findings align with the i*V*_OC_ and *V*_OC_ measurements of the triple‐junction solar cell. Also, improvements in *FF* are expected due to higher hole concentrations in the perovskite bulk toward the HTL.

Furthermore, we explore all‐perovskite tandem solar cells, where the ITO and ZTO forms an interface with the PEDOT:PSS/LBG perovskite subcell.

**Figure 10:**
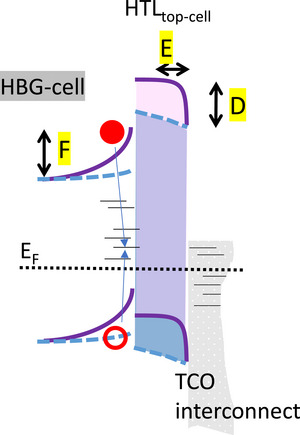
Simplified band diagram for two HTLs with different screening length. D: Fully screened band bending. E: screening length < HTL thickness. F: reduced selective contact.

**Figure** **10** presents a simplified band diagram of HBG perovskite/HTL interconnection with different HTL screening lengths, in blue and purple. It is known that the work function difference between the HTL and TCO interconnect induces a parasitic band bending. While the purple HTL can fully screen this band bending (screening length E < HTL thickness), for the blue HTL the screening length extends beyond the HTL thickness into the perovskite absorber. Which in turn, means that the induced hole selective junction F in the LBG perovskite is reduced, facilitating minority charge carrier recombination.

**Figure 11:**
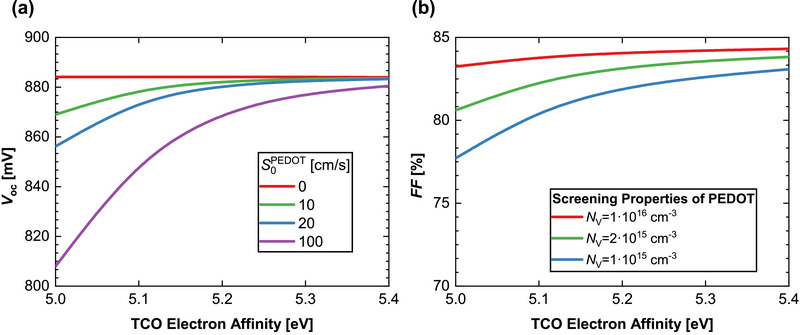
a) V_OC_ of the LBG perovskite as a function of TCO electron affinity for varying surface recombination velocities (S_0,_^PEDOT^) at the PEDOT:PSS/perovskite interface. b) Fill factor (FF) as a function of TCO electron affinity for different hole density of states (*N*_V_) in the 20 nm‐thick PEDOT layer.

**Figure** **11**a depicts the *V*_OC_ of the LBG perovskite as a function of TCO electron affinity for various surface recombination velocities (*S*_0_^PEDOT^) at the PEDOT:PSS/perovskite interface. Notably, under optimal surface passivation (*S*_0_ = 0 cm s^−1^, shown in red), the *V*_OC_ remains independent of TCO electron affinity and effective work function at the hole contact. However, even minor surface recombination velocities (*S*_0_ > 0) lead to a *V*_OC_ reduction that becomes more prominent for lower electron affinities. This effect can be attributed to the increased SRH recombination at the interface due to diminished field‐effect passivation. In this model, selectivity losses are negligible, resulting in i*V*_OC_ = *V*_OC_ throughout Figure 11a (i*V*_OC_, not shown).

Figure 11b illustrates the primary effect on the *FF* as a function of TCO electron affinity for various screening properties^[^ ^32^ ^]^ of the 20 nm‐thick PEDOT:PSS layer. The screening properties are assessed by varying the PEDOT hole density of states (*N*_V_).^[^ ^33^ ^]^ For high *N*_V_ of 10^16^ cm^−3^ (red line), the PEDOT effectively screens most of the parasitic band bending, rendering *FF* relatively independent of the TCO work function. It is known that the mobility values of the PEDOT:PSS used (PEDOT AI 4083) are in the order of 10^−4^ cm^2^ V^−1^ s^−1^.^[^ ^34^, ^35^ ^]^ Taking into consideration that conductivity is in the order of (10^−4^−10^−3^) S cm^−1^, we expect a hole density of n = 10^16^ cm^−3^. Based on this calculation, the simulations have been conducted. In contrast, for lower *N*_V_ values, the screening length of the PEDOT layer increases, resulting in diminished screening effects that are reflected in a greater dependency of *FF* on the TCO work function. PEDOT:PSS is the state‐of the art HTL material in all‐perovskite tandem cells, but it is not a perfect contact (S_0_>0). Thus, pairing it with a TCO of high work function can increase both *V*_OC_ and *FF*.

### 2.5 Perspective

**Figure 12:**
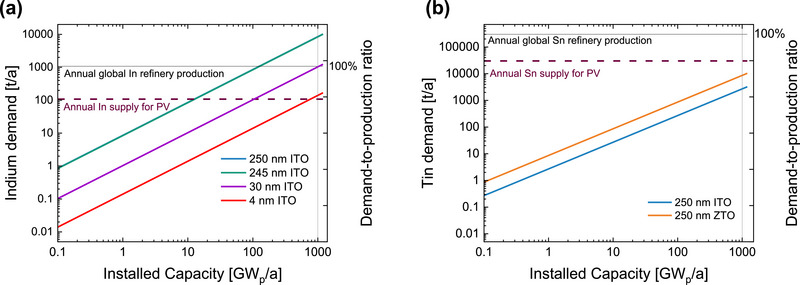
Demand versus the perovskite/perovskite/silicon solar cell installed capacity for a) indium material based on 250 nm ITO as reference and 245 nm ITO (blue and green lines almost overlap) and b) tin material based on 250 nm ITO or ZTO layers. The global refinery production for In and Sn are shown by solid line. The approximation of the supply allocated to photovoltaics (PV) (10% of the global production) is marked by dash line. The demand‐to‐production ratio compares the In or Sn demand for PV deployment with their global production in 2022. Global production values for tin is based on^[^ ^36^ ^]^ and for indium on.^[^ ^12^ ^]^

The successful replacement of ITO with ZTO is an important step toward reducing the consumption of indium. **Figure** **12** presents the indium and tin demand for perovskite/perovskite/silicon solar cell deployment on various scales, based on the TCO layer thickness. The details on the material estimation can be found in the Note S1 (Supporting Information). We note that our analysis prioritizes the assessment of resource certainty; bound reserves represent a more predictable quantity than economic factors that are subject to rapid change due to learning curves and process optimization.

Considering a perovskite‐based multijunction solar cell with 250 nm ITO (sum of the ITO layers used in a perovskite/perovskite/silicon triple‐junction solar cell), the indium consumption would be 8.6 mg W_p_^−1^. With this, by around 126 GW_p_ of installed capacity, the indium demand already reaches the annual global refinery of indium production (1080 t a^−1^).^[^ ^12^ ^]^ This represents a considerable risk for upscaling this technology. Replacing the ITO RL with ZTO reduces the demand to 8.4 mg W_p_^−1^, which only has a marginal impact on the demand‐to‐production ratio, because of the remaining indium‐based layers in the rest of the cell structure. To ensure that at 1 TW annual installed capacity, the indium demand stays below the indium production, the demand must be reduced to ≈1 mg W_p_^−1^, which corresponds to around 30 nm ITO in the solar cell structure (Figure 12a). The share of global indium supply allocated to PV will reduce to around 10% of the total indium refinery. To ensure that the indium demand for PV raises low supply concerns, the indium consumption should be further reduced: for example, up to ≈0.1 mg W_p_^−1^ that corresponds to less than 4 nm ITO in the solar cell structure. Hence, future work should focus on replacing other indium dependent layers, especially in silicon heterojunction bottom cells where the back and top TCOs are based on indium. Replacing all ITO layers might not be straightforward, especially for layers other than RLs as the requirements differ. For example, employment of the layers with high lateral resistivity, such as ZTO as the top electrode is an important challenge that needs to be addressed. Furthermore, replacing ITO with ZTO increases the tin demand since the target material has a higher tin content. However, looking into the criticality of tin, this replacement does not impose a shortage risk as the demand‐to‐production ratio stays below 100% (Figure 12b).

**Figure 13:**
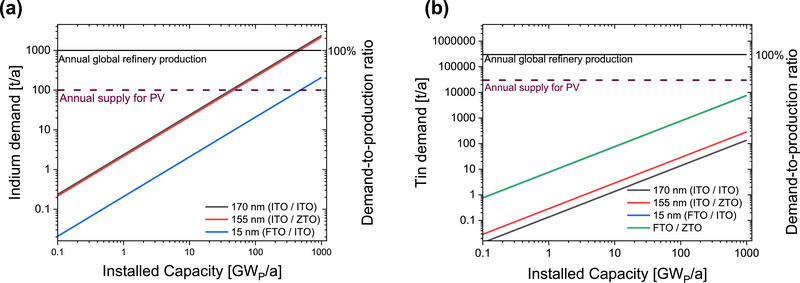
a) Installed PV capacity versus indium demand and indium demand to production ratio. Three cases are investigated. 170 nm (ITO/ITO) corresponds to the baseline device, 155 nm (ITO/ZTO) corresponds to using ZTO as RL and 15 nm (FTO/ITO) corresponds to using FTO as top TCO and ITO as RL. b) Installed PV capacity versus tin demand and tin demand to production ratio (blue and green lines almost overlap). Global production values for tin is based on^[^ ^36^ ^]^ and for indium on.^[^ ^12^ ^]^

In a similar manner, it would not be possible for the all‐perovskite tandem solar cell technology, to reach 1 TW_p_ using ITO‐based electrodes and RLs as can be seen from **Figure** **13**a. When ITO is used as both top TCO and RL, the total indium consumption far exceeds both the annual indium supply, and the annual indium supply allocated specifically for PV. When ITO is substituted with ZTO as RL, 155 nm of total ITO remains, which again does not allow for smooth upscaling of the technology. If FTO is used as top TCO, to substitute the majority of ITO at the front contact (155 nm), the material consumption would still exceed the annual ITO supply for PV. The material requirements could be solved by exchanging all ITO from the device with FTO and ZTO as shown in Figure 13b. The utilization of FTO and ZTO removes demand on indium reserves or production, while the demand for tin remains bellow both the annual tin supply and the annual supply for PV. We demonstrate the proof of concept of such an ITO‐free all‐perovskite tandem solar cell in Figure S16 (Supporting Information), where *jV* curves (measured with a spectrally adjusted spectrum) of such devices are provided. It is shown that the ITO‐free technology outperforms the conventional ITO‐based devices while solving the material criticality (information regarding the demand to production ratio and bound‐reserve ratio can be found in the Figure S17, Supporting Information).

## 3 Conclusion

In this work, a zinc tin oxide (ZTO) layer deposited via a scalable sputtering process is introduced as a novel indium‐free recombination layer (RL) between two perovskite subcells. By implementing this layer in both perovskite/perovskite/silicon triple‐junction and all‐perovskite tandem solar cells we have shown the compatibility of this layer with different materials and device structures. In addition, in both structures, replacing ITO with ZTO resulted in enhanced performance governed by *V*_OC_ improvements.

In the case of triple‐junction solar cells, where the 2PACz and high‐bandgap (HBG) perovskite layers are processed onto the RL, the *V*_OC_ improvement is >50 mV. The simulation results attribute the *V*_OC_ improvement to a higher effective work function when using SAM/ZTO. The higher work function potentially arises from lower doping concentration of ZTO, or the difference in TCO electron affinity. In addition, it is known that SAM properties such as dipole strength and coverage play a major role in the effective work function. When replacing ITO with ZTO, one of these factors or a combination of them leads to the observed *V*_OC_ improvement. Notably, while a significant increase in *V*_OC_ was achieved, there was only a slight rise in the internal voltage (i*V*_OC_). This shows a reduction in selectivity losses, as showcased in the simulation when transitioning from higher to lower doping concentrations. This is well aligned with the electron concentrations measured for ITO and ZTO.

All‐perovskite tandem solar cells, where the PEDOT:PSS and low‐bandgap (LBG) perovskite layers are processed onto the RL, have shown an increase in the *V*_OC_ of ≈30 mV. At the same time, the *FF* of the ZTO containing devices is increased comparably to the ITO‐based devices. From PL measurements, we show that when ZTO is utilized, the i*V*_OC_ of the LBG absorber is increased by 30 mV, due to a decrease in non‐radiative recombination, which contributes to an increased *V*_OC_. This can be explained by the higher ZTO work function that makes the ZTO a better hole contact than ITO. Our experimental findings are further supported by simulation results, that showcase the impact of the TCO work function on the *V*_OC_ for a non‐ideal HTL.

The champion perovskite/perovskite/silicon triple‐junction solar cell with ZTO, based on this new recombination layer produced by scalable sputter deposition, showed an excellent *V*_OC_ of 3.10 V, a *FF* of 84.9%, a *j*_SC_ of 8.3 mA cm^−2,^ and a *PCE* of 21.9%. Additionally, we showcase the first ITO‐free all‐perovskite tandem solar cell, which utilizes FTO as front electrode and ZTO as RL with *j*_SC_ of 14.7 mA cm^−2^, *FF* of 68.0%, *V*_OC_ of 2.07 V, and *PCE* of 21.0%. Our work paves the way for upscaling the perovskite multijunction technologies efficiently and sustainably.

## 4 Experimental Section

### Solar Cell Fabrication—Solutions Preparation

*High‐Bandgap Precursor Solution In All‐Perovskite Tandem*: Cs_0.17_FA_0.83_Pb(I_0.60_Br_0.40_)_3_: For the 1.2 m HBG perovskite solution 53.0 mg of CsI, 171.3 mg of FAI, 221.3 mg of PbBr_2,_ and 264.2 mg of PbI_2_ were mixed in a 4:1 ratio of DMF:DMSO and left to stir for 2 hours. Then the solution was filtered with 0.22 µm filter and 1 mg mL^−1^ of Urea was added.

*High‐Bandgap Precursor Solution in Triple‐Junction Solar Cell* ^37^ ^]^:Cs_0.20_FA_0.71_MA_0.09_Pb(I_0.64_Br_0.27_Cl_0.09_)_3_: First 1.0 m perovskite was prepared by dissolving 134.1 mg of FAI, 57.1 mg of CsI, 253.6 mg of PbI_2_, and 192.7 mg of PbBr_2_ (with 17% PbBr_2_ excess) in DMF: DMSO (3:1 volume ratio) and stirring overnight at room temperature. On the following day 6.8 mg MACl and 27.8 mg PbCl_2_ were added to the solution and stirred at 60 °C for more than 2 hours. The final solution was then diluted to 0.9 m.

*Middle‐Bandgap Precursor Solution in Triple‐Junction Solar Cell*: Cs_0.05_(FA_0.90_MA_0.10_)_0.95_Pb(I_0.95_Br_0.05_)_3_: For the preparation of 1.2 m perovskite solution, 176.4 mg of FAI, 12.8 mg of MABr, 15.6 mg of CsI, 12.2 mg of PbBr_2_ and 543.4.5 mg of PbI_2_ (1% Pb excess) were dissolved in DMF: DMSO (4:1) solvent mixture and stirred overnight.

*Low‐Bandgap Precursor Solution in All‐Perovskite Tandem*: FA_0.70_MA_0.30_Pb_0.50_Sn_0.50_I_3_: For the 2.0 m LBG perovskite precursor solution 461.0 mg of PbI_2_, 372.5 mg of SnI_2_, 240.8 mg of FAI and 95.4 mg of MAI were mixed in 4:1 DMF:DMSO solution and left to stir overnight. Additionally, 15.7 mg of SnF_2_, 3.2 mg of Pb(SCN)_2_, 1.3 mg of MACl, 2.2 mg of GlyHCl and 5 mg of Sn powder were added.

### Solar Cell Fabrication—Silicon Bottom Cells

Silicon bottom cells were fabricated using 250 µm‐thick p‐type float‐zone silicon wafers (bright etched, Siltronic) with a base resistivity of about 1 Ω cm. A pyramidal texture was etched onto the rear side only using potassium hydroxide (KOH) using an industrial tool (SINGULUS SILEX). After etching off the silicon oxide thin film protecting the planar front side during texturing, the wafers were subjected to O_3_‐based wet‐chemical cleaning before transferring them to the plasma‐enhanced chemical vapor deposition (PECVD) cluster tool (INDEOtec Octopus II) where a stack of intrinsic/doped amorphous silicon passivation layers was deposited on both sides. The thickness of *n*‐doped / undoped a‐Si:H and *p*‐doped / undoped a‐Si:H for the planar front and textured rear surfaces was ≈19 and 27 nm, respectively‐ The PECVD process was carried out in a parallel‐plate reactor, powered at 13.56 MHz and at a temperature of 200 °C, using a mixture of silane (SiH_4_), hydrogen (H_2_), phosphine (PH_3_), and trimethylborane (TMB). Next, a ≈20 nm recombination layer was formed through a 1 cm^2^ shadow mask on the front via direct‐current sputtered ITO (In_2_O_3_/SnO_2_ = 97/3 wt.%,) in an VON ARDENNE SCALA in‐line tool using a mixture of oxygen and argon. The rear contact was completed by ≈190 nm of ITO followed by ≈1000 nm of Ag on the full area (For both the front and rear TCO deposition an oxygen to argon ratio of 5.3% was used). Finally, the wafers were laser‐scribed to 2.5 × 2.5 cm^2^ substrates, with the 1 cm × 1 cm ITO pad in the middle defining the active area. The thin film thicknesses above correspond to films co‐deposited on planar silicon for process control. Hence, the actual thickness on textured samples is reduced due to increased surface area, depending on the deposition technique.

Perovskite/perovskite dual‐junction cells were fabricated on ohmic silicon substrates. These silicon architectures are meant to provide the same substrate properties (texture, rec. TCO) to not alter top cell grows, but are lacking the p/n‐junction of the bottom cell. To function as an ohmic substrate, n‐type float‐zone silicon wafers with a base resistivity of 1 Ωcm were used instead of p‐type wafers and at the rear side the *p*‐doped is replaced by *n*‐doped a‐Si: H. The rear side TCO is omitted, and a titanium Ag stack is deposited by evaporation.

### Solar Cell Fabrication—All‐Perovskite Solar Cells

*On Glass Substrate*: 30 Ohm cm^−2^ prepatterned glass/ITO substrates were cleaned in an ultrasonic bath of acetone, isopropanol and water for 5 min each. After drying with N_2_ they have undergone Ultraviolet (UV)‐O_3_ treatment for 20 min. 100 µL of 1 mm of Me‐4PACz in ethanol was statically spin‐coated on the substrate and annealed for 10 min at 100 °C. Aluminum oxide (Al_2_O*_3_*) from Sigma–Aldrich (Lot number MKCJ4233) was diluted further into 1:500 AlO*_x_*:IPA and kept stirring until use. Then, 100 µL of Al_2_O*_3_* was coated dynamically at 4000 rpm with 1000 rpm acceleration for 30 s and annealed for 2 minutes at 100 °C. After the substrates were cooled down to room temperature the perovskite precursor solution was processed on them. The HBG absorber was coated via a two‐step deposition that consists of a 2000 rpm with 1000 rpm acceleration for 10 seconds program followed by a 6000 rpm with 2000 rpm acceleration for 40 second step. 20 second prior to the end of the second program 200 µL of antisolvent chlorobenzene were dynamically deposited. Then the devices are annealed at 100 °C for 15 minutes. When the substrates are cooled down to room temperature, 100 µL of PCBM solution (10 mg mL^−1^ in chloroform) were dynamically dispensed at 4000 rpm for 30 seconds. Then, 30 nm of SnO*_x_* were deposited via an ALD deposition process. For the sputtering of both RL a pixelated shadow mask was used, featuring the layout of the final solar cell area. Deposition of ITO was done at an Oxford cluster tool while ZTO was sputtered on VISTARIS 600 industrial compatible tool. On the sputtered TCOs, PEDOT:PSS was statically spin‐coated on the substrates at 3000 rpm for 60 seconds and annealed at 140 °C for 10 minutes. Later, the LBG precursor solution was statically spin‐coated with a two‐step process of 2000 rpm with 1000 rpm acceleration for 10 seconds program followed by a 4000 rpm with 2000 rpm acceleration for 40 seconds. 20 seconds prior to the end of the second program 200 µL of antisolvent Anisole were dynamically deposited. Then the devices were annealed at 100 °C for 10 minutes. As the next step, 15 nm of C_60_ were thermally evaporated and then 20 nm of SnO*_x_* was deposited via ALD. After structuring 100 nm of silver were thermally evaporated.

### Solar Cell Fabrication—MBG Perovskite/HBG Perovskite Dual‐Junction Solar Cells

*On Ohmic Silicon Substrate*: Silicon substrates were first cleaned by spin‐coating 200 µL ethanol following by 15 minutes UV‐Ozone treatment. The clean substrates were then transferred to a nitrogen filled glovebox. 120 µL of PTAA with a concentration of 3 mg mL^−1^ dissolved in Toluene was spin‐coated with a 6000 rpm for 30 s program. The substrates were then annealed on a preheated hotplate for 10 minutes at 100 °C. To improve the wetting, 50 µL of PFN was spin‐coated at 5000 rpm for 20 seconds with no further annealing. For deposition of the middle perovskite layer, 150 µL solution was statically spin‐coated with a 4000 rpm for 35 seconds program and 300 µL ethyl acetate as antisolvent was dropped on the spinning substrates 25 seconds before the end of the program. The devices were then annealed for 30 minutes at 100 °C. As the ETL, 15 nm of C_60_ was thermally evaporated. 30 nm SnO*_x_* was deposited on top as a buffer layer to prevent sputter damage and act as solvent barrier layer. For reference samples, 5 nm of ITO was deposited through a shadow mask with an 11 mm by 11 mm opening. The sputtering was done using an Oxford instruments cluster tool. For the target samples, 15 nm of ZTO was sputtered using VISTARIS 600 and utilizing the same shadow mask. The ZTO target was provided by GfE.

Afterward, the samples were transferred to a glovebox for processing of the top perovskite cell. 130 µL of 2PACz with 0.3 mg mL^−1^ concentration dissolved in ethanol were spin‐coated at 3000 rpm for 30 seconds. The samples were then annealed for 10 minutes at 100 °C. 150 µL of HBG perovskite solution were dynamically spin‐coated at 4000 rpm for 35 seconds with a ramp of 3 seconds. N_2_ was blown on the spinning substrate from 20 to 10 seconds to the end of the program. An annealing step for 30 minutes at 100 °C followed. For the passivation layer, 100 µL of the PI solution (0.4 mg mL^−1^ dissolved in IPA) was spin‐coated on the samples at 5000 rpm for 30 seconds with a ramp of 4 seconds and annealed for 10 minutes at 100 °C. Afterward 100 µL of IPA was spin‐coated using the same spin‐coating program. Samples were annealed for 5 minutes at 100 °C after the washing step. Similar to the middle cell, 15 nm of C_60_ were evaporated as ETL. A 20 nm thick SnO*_x_* layer was then deposited by ALD. As top TCO, 25 nm of ITO was sputtered through the same shadow mask as the recombination layer. Metallization was done by evaporation of 200 nm silver. Finally, 100 nm of magnesium fluoride was evaporated as antireflection coating.

*Triple‐Junction Solar Cells*: The flat front and texture rear side heterojunction silicon bottom cell was used as described above. The rest of the processing of the triple‐junction solar cells was similar to the perovskite/perovskite solar cells on ohmic silicon substrates and according to.^[^ ^37^ ^]^

### Characterization—IV Measurements

For the multijunction solar cells on silicon substrates, the *jV* measurements were performed with a Wavelabs SINUS 220 light‐emitting diode (LED)‐based solar simulator containing 20 different LED channels. For both dual‐junction and triple‐junction solar cells, the spectral responses (SR) were measured prior to the *jV* measurement. The spectrum was then calculated using the relative SRs according to the procedure described in.^[^ ^38^ ^]^ This ensures that the spectrum is correctly adjusted such that the photocurrent generated by each subcell under the simulator spectrum matches the photocurrent generated under the AM1.5 g spectrum. The *jV* curves were then measured starting from −100 mV up to 2400 mV for dual‐junction solar cells and up to 3100 mV for triple‐junction solar cells with a voltage step of 6 mV and a scan rate of 60 mV s^−1^ first in forward, then in reverse scan direction. The temperature of the measurement chuck was set to 25 °C.

For the all‐perovskite tandem solar cells, the *jV* were measured using a Keithley 2400 source meter under illumination from a solar simulator (Newport, Class AAA) with a light intensity of 100 mW cm^−2^ (checked with a calibrated reference solar cell from CalLab PV Cells / PV Modules ‐ Fraunhofer ISE). Spectral adjustment was not considered leading to large uncertainties in *j*_SC_ and *FF*. *jV* curves were measured in a nitrogen atmosphere with a scanning rate of 100 mV s^−1^ (voltage step of 10 mV and delay time of 100 ms).

### Characterization—*EQE* Measurements

EQEs were measured with an in‐house setup. A Xenon lamp is used as light source. The light is chopped at ≈133 Hz and directed through a double grating monochromator to produce monochromatic light. A transimpedance amplifier provides bias voltage during the measurements and amplifies the signal, which is then detected by a lock‐in amplifier. The EQE of the devices was measured according to the procedure described in reference.^[^ ^4^, ^39^ ^]^ For the triple‐junction measurements, selective infrared and red LEDs (850 nm and 740 nm, respectively) were used for the measurement of the perovskite top cell. For the middle cell measurement, blue (465 nm) and infrared LEDs were employed. The silicon bottom cell was measured under selective blue and red LEDs. A bias voltage was applied to the device for each subcell measurement, following the international standard procedure.^[^ ^39^ ^]^ For the dual‐junction cells on ohmic silicon cells, blue and red LEDs were used to measure the MBG perovskite and HBG perovskite, respectively. For the all‐perovskite tandem solar cells on glass, blue and IR LEDs were used to measure the LBG and HBG bandgap perovskites respectively. The EQE was recorded in 10 nm increments and the temperature was maintained at 25 °C.

### Characterization—i*V*_OC_ Imaging

i*V*_OC_ images were acquired using a commercial PL imaging system from Intego GmbH that was developed at Fraunhofer ISE. Laser with peak wavelength at 450 was used to illuminate the HBG perovskite subcells. 808 nm laser was used to illuminate silicon bottom cell and LBG perovskite subcell. The illumination intensity was adjusted to the AM1.5g conditions using the relative EQEs according to.^[^ ^25^ ^]^ The MBG perovskite subcell was illuminated selectively using an LED with emission peak wavelength at 730 nm. This light source was not calibrated to the AM1.5g spectrum. Optical bandpass filters were used to separate the luminescence signal of the different subcells before it was captured in a silicon CCD camera. Further details on the i*V*_OC_ imaging method are available elsewhere.^[^ ^26^ ^]^

### Characterization—*XRD* Measurements

A Bruker D8 Advance tool with a copper anode X‐ray source (*K*_α_) was used to measure the X‐ray diffraction (XRD) of the layers. Measurements were recorded in the 2*θ* range of 5–60° with a step size of 0.02°. DIFFRAC.EVA software is used for data analysis.

### Characterization—*UV–Vis* Measurements

Reflectance and transmittance measurements were carried out using a Lambda 950 spectrophotometer from PerkinElmer equipped with an integrating sphere. Samples were measured in a wavelength range of 250 – 1200 nm with a 2 nm step size.

### Characterization—*AFM* Measurements

Atomic force microscopy (AFM) measurements were performed in PeakForce Tapping mode using a Dimension Edge AFM (Bruker) with SCANASYST‐AIR probes (Bruker). AFM images were recorded on area of 2 µm × 2 µm, using a resolution of 1024 × 1024 pixels.

### Characterization—*SEM* Measurements

SEM pictures were taken using a Schottky emission scanning electron microscope (Zeiss, Auriga 60) at 5 kV.

### Characterization—Kelvin Probe Measurements

Kelvin Probe measurements were performed using a UHVKP 4.5 system bought by KP TECHNOLOGY. The Kelvin Probe system is integrated in the Oxford Clustertool to ensure vacuum conditions during measuring.

### Characterization—Kelvin Probe Force Microscopy (KPFM) Measurements

KPFM measurements performed using a Veeco Dimension Icon AFM. The SCM‐PIT‐v2 probe, coated with Pt/Ir, with 75 kHz cantilever frequency and 4 N m^−1^ force constant was used for both topography and contact potential difference (CPD) mapping in a dual‐pass mode. The measurements were conducted in air. The probe work function was calibrated using a HOPG reference. The work functions of samples were calculated according to the equation: *V*_CPD_ = (WF_probe_ – WF_sample_)/e, where V_CPD_ is the measured CPD, WF_probe_ is the work function of the probe, WF_sample_ is the work function of the sample, and e is an elementary charge.

### Characterization—Photoelectron Spectroscopy in Air (PESA) Measurements

PESA measurements were conducted in air with a Riken Keiki AC‐2 spectrometer, equipped with a D2 lamp and a grating monochromator. The scan step was 0.05 eV, counting time 10 s, and incident UV intensity varied from 20 nW for TCOs with SAMs and PEDOT:PSS to 80 nW for bare TCOs.

### Characterization—Simulations

The opto‐electrical simulation model was set up in Sentaurus TCAD^[^ ^40^ ^]^ that is capable to describe the detailed physics of a perovskite/silicon tandem solar cells. The model was experimentally validated in several previous publications regarding two‐terminal tandem configurations^[^ ^28^, ^41^ ^]^ and has been extended to a triple‐junction simulation model in.^[^ ^29^ ^]^ Details on the SAM model can be found in Landgraf et al.^[^ ^30^ ^]^

## Conflict of Interest

The authors declare no conflict of interest.

## Supporting information



Supporting Information

## Data Availability

The data that support the findings of this study are available from the corresponding author upon reasonable request.
