# Supplementary material for: Indium‐Free Recombination Layer for Perovskite‐Based Multijunction‐Solar‐Cells‐ with Improved Performance Using Sputtered Zinc Tin Oxide
Source: Small. 2025 Nov 18;21(50):e11646. doi: 10.1002/smll.202511646 (PMC12710185; doi:10.1002/smll.202511646)
Supplement: Supplementary file 1 — Supporting Information [file SMLL-21-e11646-s001.docx]

**Indium-free recombination layer for perovskite-based multi‑junction solar cells with improved performance using sputtered zinc tin oxide**

Maryamsadat Heydarian^1*^, Georgios Loukeris^1,2,3*^, Martin Bivour^1^, Christoph Messmer^1,4^, Minasadat Heydarian^1,4^, Oliver Fischer^1,4^, Clemens Baretzky^1,4^, Alexander J. Bett^1^, Estelle Gevais^1^, Muhammad Fareed U Din Masood^1,5^, Sofiia Kosar^6^, Stefaan De Wolf^6^, Florian Schindler^1^, Martin C. Schubert^1^, Markus Kohlstädt^1,2,3^, Juliane Borchert^1,4^, Uli Würfel^1,2^, Patricia S. C. Schulze^1^, Andreas W. Bett^1,3^, Stefan W. Glunz^1,2,4^

^1^ Fraunhofer Institute for Solar Energy Systems, Heidenhofstr. 2, 79110 Freiburg, Germany

^2^ Freiburg Materials Research Center FMF, University of Freiburg, Stefan-Meier-Str. 21, 79104 Freiburg, Germany

^3^ Institute of Physics, University of Freiburg, Herman-Herder-Straße 3, 79104 Freiburg, Germany

^4^ Chair for Photovoltaic Energy Conversion, Department of Sustainable Systems Engineering (INATECH), University of Freiburg, Emmy-Noether-Str. 2, 79110 Freiburg, Germany

^5^Department of Physics, Technical University of Munich, James-Franck-Str. 1, 85748 Garching, Germany

^6^ Center for Renewable Energy and Storage Technologies (CREST), Physical Science and Engineering Division (PSE), King Abdullah University of Science and Technology (KAUST), Thuwal 23955-6900, Kingdom of Saudi Arabia

E-mail: [Maryamsadat.Heydarian@ise.fraunhofer.de](mailto:Maryamsadat.Heydarian@ise.fraunhofer.de), georgios.loukeris@ise.fraunhofer.de

Keywords: photovoltaics, recombination layer, tandem solar cells, all-perovskite tandem solar cell, triple-junction solar cell

^*^ Maryamsadat Heydarian and Georgios Loukeris contributed equally to this work

Figure S1 XRD patterns of ITO and ZTO layers deposited on glass substrates with or without annealing at 300 °C for 5 minutes. As-deposited ITO and ZTO show no peaks prior to the annealing. After the annealing, XRD of the ITO shows peaks, indicating crystallization of ITO.

Figure S2 Tauc plot with bandgap estimation (from intercept of the linear fit with the x-axis) for ITO and ZTO layer deposited on a glass substrate. ZTO shows ~ 0.1 eV higher bandgap than the ITO.


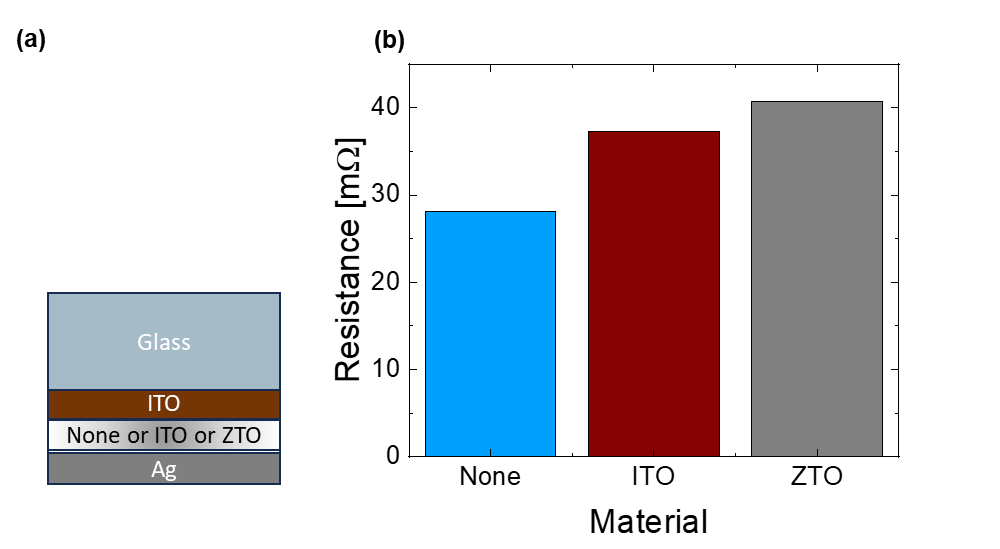


Figure S3 (a) Layer stack used for the resistance measurements. (b) Resistance measurements conducted on test structures consisting of glass/ITO/Ag, glass/ITO/ITO/Ag and glass/ITO/ZTO/Ag. Ag serves as top electrode while the first ITO layer serves as bottom electrode.

Figure S4 Champion MBG perovskite/HBG perovskite dual-junction solar cells on ohmic silicon substrates with ZTO or ITO as recombination layers.

Figure S5 Normalized photoluminescence (PL) spectra of HBG perovskite solar cells under constant illumination. The measurement was performed on perovskite single-junction devices with the device structure ohmic silicon/ITO/2PACz/1.83 eV perovskite/C_60_/SnO_X_/ITO/Ag/MgF_2_ and ohmic silicon/ZTO/2PACz/1.83 eV perovskite/C_60_/SnO_X_/ITO/Ag/MgF_2_.

Figure S6 The EQE curves of (a) MBG perovskite/HBG perovskite dual-junction solar cells on ohmic silicon substrate and (b) perovskite/perovskite/silicon triple-junction solar cells with ITO and ZTO recombination layers between the perovskite subcells.

Figure S7 (a) Schematic of the perovskite/perovskite/silicon triple-junction solar cell structure. The illumination and processing directions are shown with yellow and black arrows, respectively. (b-e) the photovoltaic parameters of the devices with ITO and ZTO as recombination layers.

Figure S8 (a) Schematic of the perovskite/perovskite/silicon triple-junction solar cell structure. The illumination and processing directions are shown with yellow and black arrows, respectively. (b-e) the photovoltaic parameters of the devices with 5 nm or 15 nm ITO as recombination layer.

Figure S9 V_OC_ evolution of perovskite/perovskite/silicon triple-junction solar cells in literature. Data sorted by year of publication. Data taken from [1–13]

Figure S10 (a) efficiency of a triple-junction solar cell with ZTO RL measured over time at fixed voltage close to maximum power point in ambient environment with relative 60% humidity. (b) The image of triple-junction solar cell with ZTO RL after keeping under ambient environment for 48 hours. (c) The measurement setup to control the humidity during the operational stability test of the triple-junction solar cell and (d) efficiency of a triple-junction solar cell with ZTO RL measured over time at fixed voltage close to maximum power point under 20% relative humidity.

Figure S11 The EQE curves of all-perovskite tandem solar cell on glass substrate with ITO and ZTO recombination layers between the perovskite subcells.

Figure S12 (a) Champion all-perovskite tandem solar cells with ITO (ITO/ITO corresponds to front TCO/RL) or (b) ZTO (ITO/ZTO corresponds to front TCO/RL) as recombination layers.


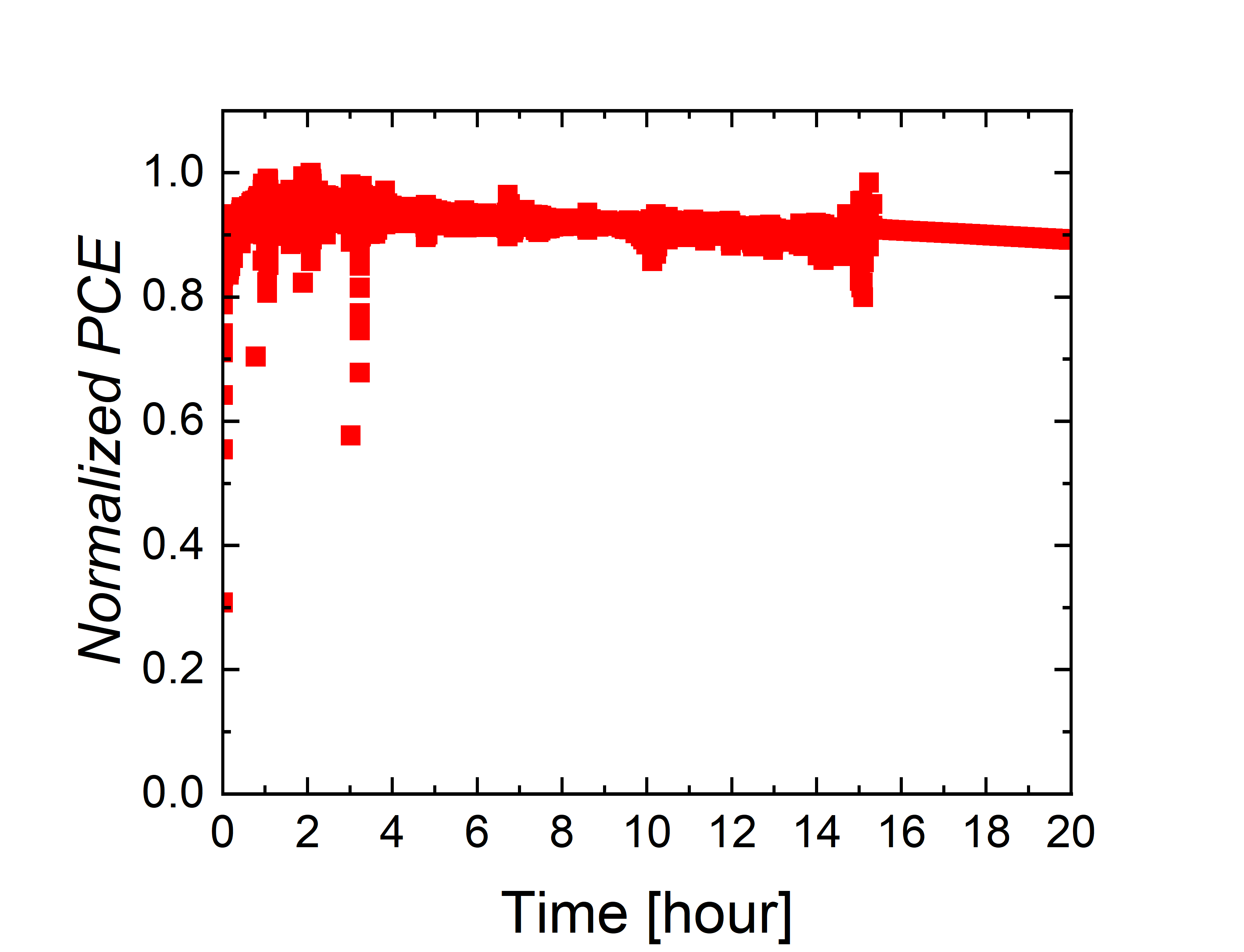


Figure S13 Efficiency of an all-perovskite tandem solar cell with ZTO RL measured over time at fixed voltage close to maximum power point in ambient environment with relative 60% humidity.

Figure S14


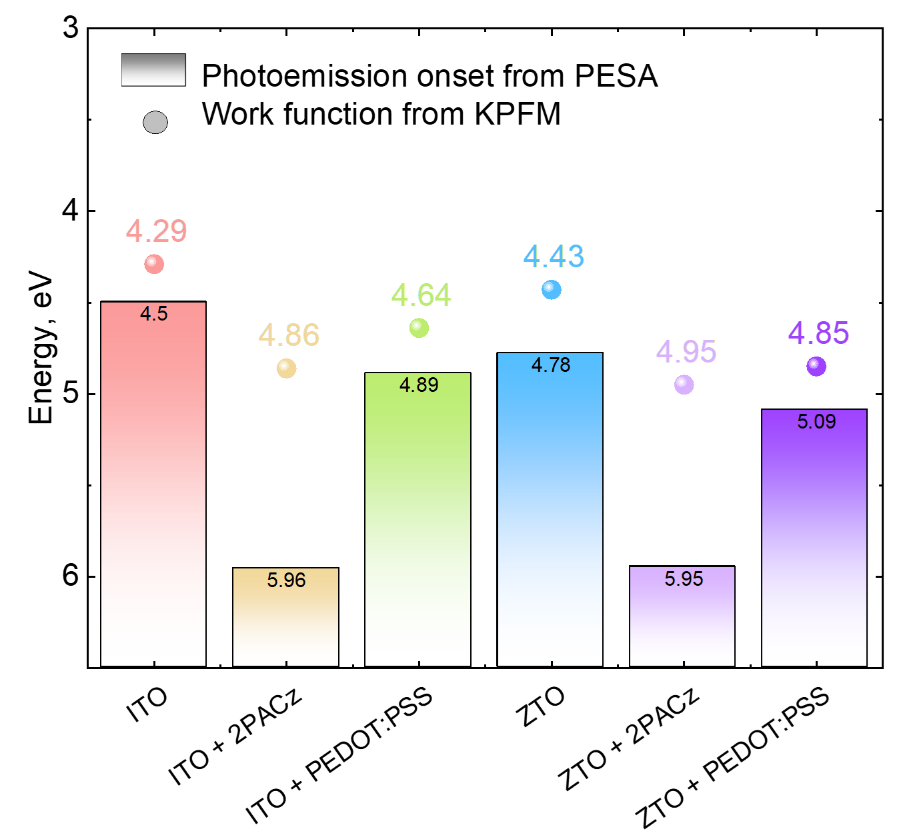


Figure S15 Photoemission onset from PESA measurements and work function estimation from KPFM measurement of ITO and ZTO with and without HTL (2PACz and PEDOT:PSS) deposition.

Figure S16 Champion all-perovskite tandem solar cells featuring FTO as front TCO. (a) while ITO (FTO/ITO corresponds to front TCO/RL) is utilised as RL and (b) when ZTO (FTO/ZTO corresponds to front TCO/RL) is used as RL, making completely indium-free all-perovskite tandem solar cell measured with adjusted spectrum. (c) MPP measurements of the device with ZTO RL and FTO front TCO. For the MPP measurement, absolute j_MPP_ value should not be considered, as measurements were carried out with a non-adjusted spectrum.

Note S1: On Scenario, description and assumption for the material analysis.

For the perovskite-based multijunction technologies, the methodology for assessing material criticality in transparent conducting oxide (TCO) interlayers, specifically evaluating the resource intensity of manufacturing processes relative to global production of constituent materials, is structured as follows [14]. To project demand for a 1 terawatt-peak (TW_p_) solar power system, the active area required is calculated using:

$$A_{\text{active}}=\frac{P_{peak, Manufactured}}{P_{\mathrm{SolarIrradiance}}\eta_{Cell}}$$

is approximately 3000 km^2.^ This provides the baseline spatial footprint, which is then used to estimate material requirements.

From the equation above, $P_{peak, Manufactured}= {10}^{12} W_{P}$, $P_{\mathrm{SolarIrradiance}} = 1 kW/m^{2}$, and $\eta_{Cell}=33\%$. This yields an active area of approximately 3000 km^2^, serving as the baseline for material demand. Material-specific densities (ITO: 7.12 g/cm^3^, FTO: 6.90 g/cm^3^, ZTO: 5.80 g/cm^3^) and stoichiometric molecular weights are used to compute elemental demand per TCO configuration, accounting for variations in composition and oxide formation. We assume a material utilization rate of 50% for sputter target efficiency. The ITO target composition is In_2_O_3_/SnO*_x_* (90/10 wt.%), while the ZTO target consists of ZnO/SnO_2_ (8/92 wt.%). In addition to ITO and ZTO, Sn consumption also arises from the SnO*_x_* buffer layer, which mitigates sputter damage. To account for this in the Sn demand calculation, we assume a 50 nm sputtered SnO*_x_* layer. However, in our processing, SnO*_x_* is deposited by atomic layer deposition (ALD) rather than sputtering, which may introduce variations in actual material consumption.

To evaluate resource risk, two metrics are employed: the Demand-Production Ratio (DPR) and Bound-Reserves Ratio (BRR). The DPR quantifies the proportion of annual global production required to meet TCO demand

$$\mathrm{DPR}\left( \% \right) = \frac{Total Element Mass_{per TWp}}{{Production of Element}_{per year}} \times100$$

while BRR compares this demand against known reserves

$$\mathrm{BRR}\left( \% \right)=\frac{Total Element Mass_{\mathrm{perTWp}}}{Known Reserves}\times100$$

Criticality is categorized as high for DPR if demand outpaces yearly production (DPR>100%) and high for BBR if yearly demand outstrips known bound reserves (BRR >100%). A medium criticality is assumed for DPR and BBR values below 100% but above 10%, a low criticality is postulated for DPR/BBR values below 10%.

Regarding all-perovskite tandem solar cells, the TCO electrode layers (e.g., ITO, FTO), thicknesses are assumed to be 130 nm for ITO, 550 nm for FTO, and 15 nm for recombination layers (for both ITO and ZTO).
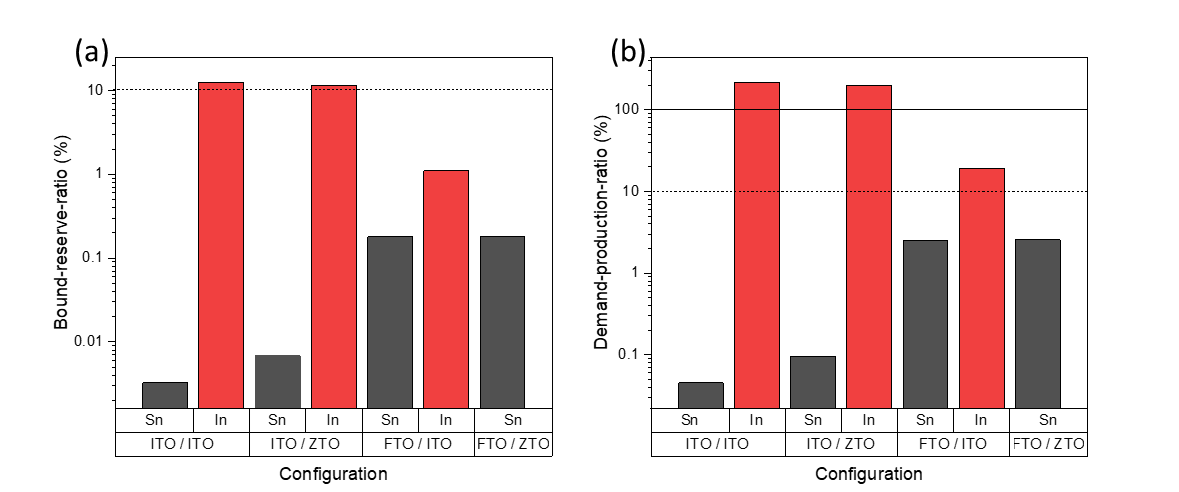


Figure S17 (a) BRR for the presented all-perovskite tandem solar cells. (b) DPR for the presented all-perovskite tandem solar cells.

The perovskite/perovskite/silicon triple-junction PV module is modeled with 72 cells, a wafer side length of 166 mm, and a module area of 2.2 m^2^. A module power output of 726 W and an efficiency of 33% are considered, which remains well below the efficiency potential of this technology [15].

Elemental supply data, such as annual production and global bound reserves, are sourced from the United States geological survey mineral commodity summary report 2025 [16]. As no data for bound indium reserves are provided in this report, this specific value is taken from [14].

Table S1. Material of interest in conjunction to annual production and bound reserves for 2025

| Material | Annual Production (t) | Bound Reserves (t) |
| --- | --- | --- |
| Indium | 1.080 | 18.800 |
| Tin | 300.000 | 4.200.000 |
| Zinc | 12.000.000 | 230.000.000 |

References

[1] J. Werner, F. Sahli, F. Fu, J.J. Diaz Leon, A. Walter, B.A. Kamino, B. Niesen, S. Nicolay, Q. Jeangros, C. Ballif, Perovskite/Perovskite/Silicon Monolithic Triple-Junction Solar Cells with a Fully Textured Design, ACS Energy Lett. 3 (2018) 2052–2058. https://doi.org/10.1021/acsenergylett.8b01165.

[2] J. Zheng, G. Wang, W. Duan, M.A. Mahmud, H. Yi, C. Xu, A. Lambertz, S. Bremner, K. Ding, S. Huang, A.W.Y. Ho-Baillie, Monolithic Perovskite–Perovskite–Silicon Triple-Junction Tandem Solar Cell with an Efficiency of over 20%, ACS Energy Lett. 7 (2022) 3003–3005. https://doi.org/10.1021/acsenergylett.2c01556.

[3] Y.J. Choi, S.Y. Lim, J.H. Park, S.G. Ji, J.Y. Kim, Atomic Layer Deposition-Free Monolithic Perovskite/Perovskite/Silicon Triple-Junction Solar Cells, ACS Energy Lett. 8 (2023) 3141–3146. https://doi.org/10.1021/acsenergylett.3c00919.

[4] M. Heydarian, M. Heydarian, A.J. Bett, M. Bivour, F. Schindler, M. Hermle, M.C. Schubert, P.S.C. Schulze, J. Borchert, S.W. Glunz, Monolithic Two-Terminal Perovskite/Perovskite/Silicon Triple-Junction Solar Cells with Open Circuit Voltage > 2.8 V, ACS Energy Lett. 8 (2023) 4186–4192. https://doi.org/10.1021/acsenergylett.3c01391.

[5] F. Xu, E. Aydin, J. Liu, E. Ugur, G.T. Harrison, L. Xu, B. Vishal, B.K. Yildirim, M. Wang, R. Ali, A.S. Subbiah, A. Yazmaciyan, S. Zhumagali, W. Yan, Y. Gao, Z. Song, C. Li, S. Fu, B. Chen, A. Ur Rehman, M. Babics, A. Razzaq, M. de Bastiani, T.G. Allen, U. Schwingenschlögl, Y. Yan, F. Laquai, E.H. Sargent, S. de Wolf, Monolithic perovskite/perovskite/silicon triple-junction solar cells with cation double displacement enabled 2.0 eV perovskites, Joule 8 (2024) 224–240. https://doi.org/10.1016/j.joule.2023.11.018.

[6] F. Li, D. Wu, Le Shang, R. Xia, H. Zhang, Z. Huang, J. Gong, L. Mao, H. Zhang, Y. Sun, T. Yang, X. Sun, Z. Feng, M. Liu, Highly Efficient Monolithic Perovskite/Perovskite/Silicon Triple-Junction Solar Cells, Adv. Mater. to be published (2024) e2311595. https://doi.org/10.1002/adma.202311595.

[7] H. Hu, S.X. An, Y. Li, S. Orooji, R. Singh, F. Schackmar, F. Laufer, Q. Jin, T. Feeney, A. Diercks, F. Gota, S. Moghadamzadeh, T. Pan, M. Rienäcker, R. Peibst, B. Abdollahi Nejand, U.W. Paetzold, Triple-junction perovskite–perovskite–silicon solar cells with power conversion efficiency of 24.4%, Energy & Environ. Sci. (2024) 2800–2814. https://doi.org/10.1039/D3EE03687A.

[8] S. Liu, Y. Lu, C. Yu, J. Li, R. Luo, R. Guo, H. Liang, X. Jia, X. Guo, Y.-D. Wang, Q. Zhou, X. Wang, S. Yang, M. Sui, P. Müller-Buschbaum, Y. Hou, Triple-junction solar cells with cyanate in ultrawide bandgap perovskites, Nature (2024). https://doi.org/10.1038/s41586-024-07226-1.

[9] T. Ye, L. Qiao, T. Wang, P. Wang, L. Zhang, R. Sun, W. Kong, M. Xu, X. Yan, J. Yang, X. Zhang, L. Ma, X. Yang, Molecular Synergistic Effect for High Efficiency Monolithic Perovskite/Perovskite/Silicon Triple‐Junction Tandem Solar Cells, Adv. Energy Mater. to be published (2024) 2402491. https://doi.org/10.1002/aenm.202402491.

[10] M. Heydarian, A. Shaji, O. Fischer, M. Günthel, O. Karalis, M. Heydarian, A.J. Bett, H. Hempel, M. Bivour, F. Schindler, M.C. Schubert, A.W. Bett, S.W. Glunz, J. Borchert, P.S.C. Schulze, 2025. Minimizing Open‐Circuit Voltage Losses in Perovskite/Perovskite/Silicon Triple‐Junction Solar Cell with Optimized Top Cell. Sol. RRL 9, 2400645. https://doi.org/10.1002/solr.202400645.

[11] Y. Shao, S. Wang, T. Luo, C. Xu, J. Liu, L. Liu, X. Dong, H. Wang, K. Wang, S. Liu, Multi-Functional Interface Engineering for Monolithic Perovskite/Perovskite/Crystalline Silicon Triple-Junction Tandem Solar Cells, ChemSusChem 18 (2025) e202402680. https://doi.org/10.1002/cssc.202402680.

[12] S.Y. Lim, Y.J. Choi, S.J. Park, G.P. Hong, J.Y. Kim, Rational Design of Medium-Bandgap Perovskite Solar Cells for Triple-Junction Si Tandems, ACS Appl. Mater. Interfaces 17 (2025) 23885–23891. https://doi.org/10.1021/acsami.4c22601.

[13] Y. Gupta, M. Heydarian, M. Heydarian, O. Er‐raji, M. Günthel, O. Fischer, C. Baretzky, P.S.C. Schulze, M. Bivour, S. De Wolf, S.W. Glunz, J. Borchert, 2025. Photostable Inorganic Perovskite Absorber via Thermal Evaporation for Monolithic Perovskite/Perovskite/Silicon Triple‐Junction Solar Cells. Prog Photovoltaics, pip.3923. https://doi.org/10.1002/pip.3923.

[14] L. Wagner, J. Suo, B. Yang, D. Bogachuk, E. Gervais, R. Pietzcker, A. Gassmann, J.C. Goldschmidt, The resource demands of multi-terawatt-scale perovskite tandem photovoltaics, Joule 8 (2024) 1142–1160. https://doi.org/10.1016/j.joule.2024.01.024.

[15] L. Restat, C. Messmer, M. Heydarian, M. Heydarian, J. Schoen, M.C. Schubert, S.W. Glunz, Optoelectrical Modeling of Perovskite/Perovskite/Silicon Triple‐Junction Solar Cells: Toward the Practical Efficiency Potential, Sol. RRL 8 (2024) 2300887. https://doi.org/10.1002/solr.202300887.

[16] National Minerals Information Center, U.S. Geological Survey Mineral Commodity Summaries 2025 Data Release (ver. 2.0, April 2025), ver. second.0, U.S. Geological Survey, 2025.
